# CORALINA: a universal method for the generation of gRNA libraries for CRISPR-based screening

**DOI:** 10.1186/s12864-016-3268-z

**Published:** 2016-11-14

**Authors:** Anna Köferle, Karolina Worf, Christopher Breunig, Valentin Baumann, Javier Herrero, Maximilian Wiesbeck, Lukas H. Hutter, Magdalena Götz, Christiane Fuchs, Stephan Beck, Stefan H. Stricker

**Affiliations:** 1 Medical Genomics, UCL Cancer Institute, University College London, 72 Huntley Street, London, WC1E 6BT UK; 2 Biostatistics, Institute of Computational Biology, Helmholtz Zentrum, German Research Center for Environmental Health, Ingolstädter Landstraße 1, 85764 Neuherberg, Germany; 3 Epigenetic Engineering, Institute of Stem Cell Research, Helmholtz Zentrum, German Research Center for Environmental Health, Ingolstädter Landstraße 1, 85764 Neuherberg, Germany; 4 Neural Stem Cells, Institute of Stem Cell Research, Helmholtz Zentrum, German Research Center for Environmental Health, Ingolstädter Landstraße 1, 85764 Neuherberg, Germany; 5 BioMedizinisches Centrum, Ludwig-Maximilian-Universität, Großhaderner Str. 9, 82152 Planegg-Martinsried, Germany; 6 Department of Biochemistry, University of Oxford, Oxford, OX1 3QU England UK; 7 Bill Lyons Informatics Centre, UCL Cancer Institute, University College London, 72 Huntley Street, London, WC1E 6BT UK

**Keywords:** gRNA library, Genome-wide, Cas9, Genetic engineering, Epigenetic engineering, Elongated protospacer, Epigenome editing

## Abstract

**Background:**

The bacterial CRISPR system is fast becoming the most popular genetic and epigenetic engineering tool due to its universal applicability and adaptability. The desire to deploy CRISPR-based methods in a large variety of species and contexts has created an urgent need for the development of easy, time- and cost-effective methods enabling large-scale screening approaches.

**Results:**

Here we describe CORALINA (comprehensive gRNA library generation through controlled nuclease activity), a method for the generation of comprehensive gRNA libraries for CRISPR-based screens. CORALINA gRNA libraries can be derived from any source of DNA without the need of complex oligonucleotide synthesis. We show the utility of CORALINA for human and mouse genomic DNA, its reproducibility in covering the most relevant genomic features including regulatory, coding and non-coding sequences and confirm the functionality of CORALINA generated gRNAs.

**Conclusions:**

The simplicity and cost-effectiveness make CORALINA suitable for any experimental system. The unprecedented sequence complexities obtainable with CORALINA libraries are a necessary pre-requisite for less biased large scale genomic and epigenomic screens.

**Electronic supplementary material:**

The online version of this article (doi:10.1186/s12864-016-3268-z) contains supplementary material, which is available to authorized users.

## Background

Reliable and efficient targeting has been the bottleneck for functional genomic and epigenomic approaches for decades. Recent research in bacterial DNA binding factors, however, provided new and highly customizable options. Most prominently used to date is the CRISPR system, which evolved in prokaryotic cells as a defense mechanism against invading phages [1]. So far the CRISPR system has been adapted to many cell types and species, where, without exception and with a high degree of specificity, robust genomic targeting of Cas9 (or modifications thereof, like the Cas9 nickase fusion protein, Cas9n [2]) has been achieved through addition of engineered guide RNA molecules (gRNAs) to those genomic DNA sequences encoded by the protospacer of the RNA sequence [3]. Endogenous Cas9 contains an endonuclease domain enabling the introduction of double strand breaks into genomic DNA [4]. Consequently, to date the most common utilization of Cas9 (or Cas9 variants) is targeted modification of the genome sequence through mutation, deletion or insertion. These approaches allow simple functional interrogation of coding, but also of noncoding regions in the genome. Promoters and enhancers [5], lncRNAs [6], miRNA response elements [7], retroviruses [8], telomeres [9] and introns [10] have already been successfully modified using CRISPR.

Genome editing with CRISPR has proven remarkably efficient [11], extending to cell types and species where this was so far not (or only insufficiently) applicable (e.g. plasmodium [12], Cryptosporidium [13], tunicates [14], wheat [15], rice [16], tomato [17], silk worms [18], C. elegans [19], beetles [20], sea lampreys [21], zebrafish [22], salmons [23], pigs [24], rats [25], goats [26], rabbits [27], and many more).

The simple and elegant concept of the CRISPR system which only requires short gRNAs for targeting, allows easy adaptation of this system to screening approaches. A series of recent studies demonstrated the use of screening approaches using pooled gRNA libraries for functional genetics and to dissect therapeutically relevant pathways (reviewed in [28]). Consequently, method development for gRNA library generation for screening is currently at the forefront of CRISPR research [29–31]. Screening libraries are, however, almost exclusively generated employing complex oligonucleotide synthesis, limiting the size and dissemination of libraries substantially due to technical restrictions and relatively high costs. Hence, for human and mouse, only gRNA libraries of limited complexity have been generated so far and none are available for other mammals.

To unleash the full potential of CRISPR-based screening for the large variety of biological model systems, new, simple, time- and cost-effective approaches are urgently needed. Likewise, to expand current screening approaches to the whole genome, including non-coding regulatory regions (e.g. those identified by ChIP or HiC approaches) [32] or to enable epigenetic screens [33], more complex gRNA library pools are required. Due to our current inability to predict which of the many genetic and epigenetic variants identified e.g. by genome- and epigenome-wide association studies are functional, we are unable to restrict gRNA libraries specifically to the corresponding relevant sites. CORALINA overcomes this limitation by generating gRNA libraries with the potential to cover virtually complete genomes in a simple, time- and cost-effective procedure.

## Methods

### Construction of guide RNA plasmids

Plasmid pMLM3636 (plasmid ID 43860) was obtained from Addgene, cut with BsmBI and a double-stranded DNA fragment, generated by annealing the two oligos MLM3636-1F (5′-ATCTTGTGGAAAGGACGAAACACCGGTTTTAGAGCTAGAAATAGCAAGTT) and MLM3636-1R (5′-AACTTGCTATTTCTAGCTCTAAAACCGGTGTTTCGTCCTTTCCACAAGAT), inserted via Gibson cloning. This yields a modified vector, pgRNA1 containing the U6 promoter, followed by the 5′G of the gRNA sequence and the scaffold sequence, but lacking the targeting sequence. For lentiviral vector construction, the vector pLKO.1 (Addgene plasmid 10878) was modified to insert the gRNA promoter and scaffolding sequence from pgRNA1. The vector was first digested with EcoRI (NEB) and AgeI (NEB). Next, the desired sequences were amplified from pgRNA1 using primers gRNA-PLKO-F (5′-TTTCTTGGGTAGTTTGCAGTTTT) and gRNA-PLKO-R (5′-ccatttgtctcgaggtcgag-TACCTCGAGCGGCCCAAGC) and inserted into PLKO.1. This vector is referred to as pgRNA-pLKO.1.

### Construction of gRNA libraries from MNase-digested genomic DNA

Human genomic DNA extracted from pooled male and female blood (250 ng/μl, provided by the Personal Genome Project UK (PGP-UK) under UCL Ethics approval 4700/001) or mouse genomic DNA (Promega) was digested with various amounts of micrococcal nuclease (NEB) to determine the optimal amount of enzyme for fragmenting genomic DNA to fragments mainly between 5 bp and 100 bp in size. The reaction setup was as follows: 1 μg genomic DNA, 1 μl 10X MNase Buffer, 0.1 μl 100X BSA in a 10 μl reaction volume was incubated with enzyme for 15 min at 37 °C. The enzyme was immediately inactivated through addition of 1 μl EGTA (500 mM). Following addition of 4 μl gel loading dye (Invitrogen), the reactions were run on a 20 % PAGE gel (Invitrogen). DNA ranging from 15 to 30 bp was excised from the gel and extracted using the Crush-and-Soak method [34]. Briefly, the gel was crushed using a sterile pipette tip and incubated in PAGE solubilisation buffer (0.5 M ammonium acetate, 10 mM magnesium acetate, 1 mM EDTA pH8) at 37 °C for 16 h and purified using a standard phenol-chloroform extraction and ethanol precipitation. Subsequently, DNA ends were repaired using the Quick Blunting kit (NEB) in a 15 μl reaction according to the manufacturer’s instructions.

A pair of adaptors for cloning the end-repaired DNA fragment into vector pgRNA-pLKO.1 via a Gibson reaction was amplified from the vector pgRNA-pLKO.1 using primers 5′-linker-F (5′-ttggaatcacacgacctgga and 5′-linker-R (5′-cggtgtttcgtcctttccac and 3′-linker-F (5′-gttttagagctagaaatagcaagttaaaata) and 3′-linker-R (5′-actcggtcatggtaagctcc) respectively. Reactions were set-up as follows: 25 μl Phusion High-Fidelity PCR Master Mix with HF Buffer (NEB), 2.5 μl of each primer (100 μM), 0.1 ng pgRNA-pLKO.1 in a total reaction volume of 50 μl. Cycling conditions: 1 cycle 98 °C for 30s, 32 cycles 98 °C for 10s, 59 °C for 10s, 72 °C for 30 s, followed by final elongation at 72 °C for 10 min. Fragments were purified using Agencourt AMPure XP beads (Beckman Coulter).

To prevent self-ligation of the linkers and ensure directionality of the ligation, the 5′ linker (689 bp) was digested with HindIII and a 600 bp fragment purified from a 1 % agarose gel using the Gel Extraction kit (QIAGEN). The 3′ linker (848 bp) was digested with SacII and the resulting 300 bp fragment gel-purified. Next, the linkers were ligated to the fragments of MNase-digested genomic DNA. 14 μl ligation reactions were set up with equimolar amounts of MNase-digested fragments (5 ng) to linkers using 1.4 μl concentrated T4 DNA ligase (NEB) and incubated at 16 °C for 16 h. Ligation reactions were directly used in nick translation, supplementing with 25 μl Long Amp Taq 2X Master Mix (NEB) and 2.5 μl primer Linker-Minus450-F (10 μM, 50-GGGCAAGTTTGTGGAATTGG) 2.5 μl primer Linker-Plus275-R (10 μM, 50- AAGTGGATCTCTGCTGTCCC) in a 50 μl reaction. Cycling conditions were 1 cycle at 72 °C for 20 min, and 3 cycles of 95 °C for 5 min, 95 °C for 15 s, 58 °C for 15 s, 72 °C for 1 min and final elongation at 72 °C for 10 min. Reactions were cleaned up with Agencourt AMPure XP (Beckman Coulter) using a sample:bead ratio of 1:1 and eluted in 40 μl of water.

Three different fragments (L1, L2 and L3) were amplified from the nick-translation product, using Phusion High-Fidelity PCR Master Mix and HF Buffer (NEB) in a 25 μl reaction supplemented with 2.5 μl of 10 μM primer and 1/16th of the purified product of nick translation (2.5 μl) as input. Primers Linker-Minus450-F and Linker-Plus275-R (sequence above) yield the full-length fragment (referred to as L1). The L2 fragment is amplified using Linker-Minus450-F and Linker-Plus160-R (5′-TCTTTCCCCTGCACTGTACC), and L3 is amplified using primers Linker-Minus150-F (5′-CCTTCACCGAGGGCCTATTT) and Linker-Plus160-R. Amplification program: 1 cycle at 98 °C for 30 s, and 16 cycles of 98 °C for 10s, 63 °C for 10 s, 72 °C for 15 s, and final elongation at 72 °C for 10 min. The L1, L2 and L3 amplification products were analyzed on a 0.8 % low-melting agarose gel. DNA of the correct size (869, 764 and 464 bp respectively) was excised from the gel and purified using the Gel Extraction kit (QIAGEN). The vector pgRNA-pLKO.1 was cut with AgeI (NEB), gel-purified and dephosphorylated using shrimp antarctic phosphatase (NEB). The three linker amplicons (L1, L2 and L3) were each cloned into the vector by Gibson assembly. Gibson assembly master mix was prepared as described [35]. 100 ng cut vector and insert in 2-fold molar excess (total volume 5 μl) were added to 15 μl of Gibson master mix and incubated at 50 °C for 1 h. A total of 16 separate reactions were set up for each type of insert and combined for purification with the Reaction Cleanup kit (QIAGEN), followed by electroporation of the entire reaction into freshly prepared electrocompetent TG1 E. coli cells with high competency (>10^10 colony-forming units per μg DNA as determined by control electroporation with pUC19 plasmid (NEB)). *E. coli* cells were allowed to recover in antibiotic-free medium for 1 h at 37 °C before plating on antibiotic containing 2TY-coated plates (Bio-assay dish with lid, 245 mm × 245 mm × 25 mm, radiation sterilized, Thermo Scientific Nunc). Following overnight incubation at 37 °C, the bacteria were harvested by scraping and the plasmid library extracted using the HiSpeed Plasmid Maxi Kit (QIAGEN).

### CORALINA library QC by sequencing

Fragments comprising the gRNA protospacer sequence were amplified from the library and Illumina adapters ligated, followed by addition of barcoded sequencing adapters by PCR and sequencing on the Illumina MiSeq platform. Please see Additional file 1: Supplementary Methods for details.

### Bioinformatic analysis

gRNA protospacer sequences were extracted from the raw reads using Cutadapt [36]. Sequences were aligned to the reference genome (human hg19 or mouse mm10 respectively) using Bowtie (version 1.1.2) without allowing mismatches [37]. gRNAs were assessed for their length, presence of a PAM sequence immediately downstream of the target site and location of the targeting site in gene, intergenic regions, and repeats, as well as GC content. To estimate the gRNA number from the sequenced samples of CORALINA libraries, we used a Bayesian approach. Please see Additional file 1: Supplementary Methods for details. The Code is available at hmgubox (https://hgmubox.helmholtz-muenchen.de:8001/d/6c6e75236e/; password: Coralina).

### Functional validation of gRNAs with extended protospacers

NGS: gRNAs aligning to a single genomic site containing an NGG PAM and possessing protospacer longer than 30 bp were randomly selected from the human L1 library. gRNAs were cloned into px458 (Addgene plasmid 48138), containing an expression cassette for S.Pyogenes Cas9-GFP. The resulting plasmids were transfected into HEK293T cells (ATCC 293 T/17, CRL-11268) and DNA harvested 48 h after transfection using the DNeasy blood and tissue kit (QIAGEN). gRNA target regions were amplified by PCR and sequenced by next-generation sequencing. Data was analysed using the CRISPR-parsr pipeline for indel scoring (https://github.com/UCL-BLIC/crispr-parsr/releases/tag/v0.2.1). For details including primer sequences please see Additional file 1: Supplementary Methods.

Flow cytometry: Additionally, gRNAs with extended protospacers targeting YFP were designed and cloned into px459 (Addgene plasmid 48139). The vectors were transiently transfected into mouse neural stem cells constitutively expressing an YFP transgene (see Additional file 1: Supplementary Methods). YFP expression was assayed 7 days after transfection by flow cytometry.

## Results

**Fig. 1:**
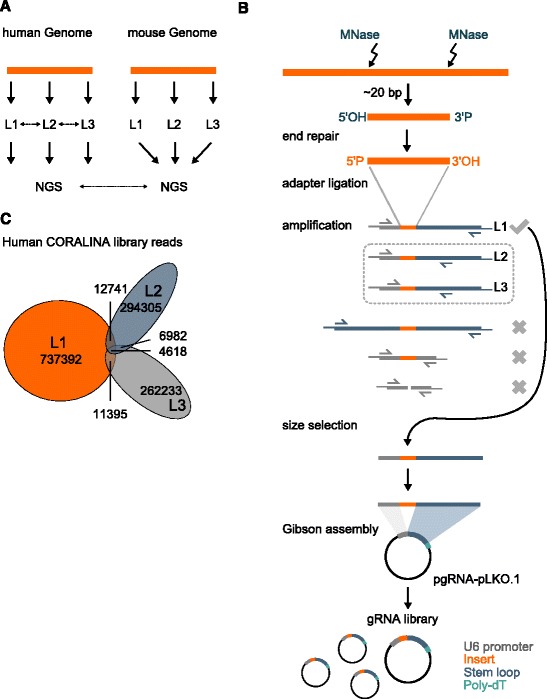
Overview of CORALINA (comprehensive gRNA library generation through controlled nuclease activity). **a** Three CORALINA libraries (L1, L2, L3) from genomic DNA of human and mouse each were generated and analyzed by NGS to compare conditions and corroborate the reproducibility of the method. **b** In principle, any type of double stranded DNA can be used as a source for CORALINA library generation. The DNA is fragmented through controlled digestion with MNase and ligated to linker oligos containing only one blunt end, but no phosphate residue. Consequently, linkers aligning in wrong orientation or to linker-oligos instead of fragments are excluded. PCR amplification allows size selection of those sequences containing two different linker sequences. Three different linker sequences (L1, L2, L3) have been used for bulk incorporation into gRNA-PLKO.1 using Gibson Assembly (p: phosphate residue; *blue*, *grey lines*: linker sequences and homology regions on gRNA-PLKO.1; *orange lines*: protospacer sequences, *small arrows*: primer sequences). **c** The analyzed representative samples of the three generated human CORALINA libraries have little sequence overlap indicating large library complexity. Shown in numbers are the unique occurrences of protospacers longer than 17 bps

To demonstrate the utility of CORALINA, we cloned multiple complex gRNA libraries (the study design is shown in Fig. 1a). While CORALINA gRNAs could be derived in principle from any source of DNA (e.g. genomic DNA from any prokaryotic or eukaryotic species, pre-digested DNA for reduced representation, immune-precipitated DNA, amplified cDNA, isolated mtDNA, ctDNA, ccfDNA or viral DNA) we used complete genomic DNA from two very large and well annotated genomes (Mus musculus and Homo sapiens) to test the optimal conditions, limits, and bottlenecks of our method. For validation and replication, we independently generated and analyzed three pooled gRNA libraries from both species to assess the reproducibility of CORALINA. In addition, CORALINA was tested for robustness to customization (e.g. different cloning strategies or delivery systems) by using different oligonucleotide linkers for the three libraries (L1, L2 and L3, Fig. 1a).

**Fig. 2:**
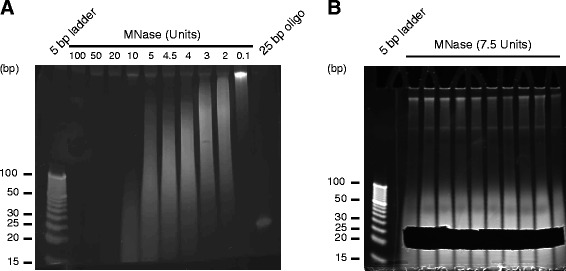
**a** Controlled digestion of genomic DNA enables generation of fragments with predictable length (<100 bp). **b** Size selection of fragments between 20 and 30 bp using polyacrylamide gels and excision

An ultimate source of gRNA libraries allowing unbiased genome-wide screening would contain all possible protospacer sequences found in the genomic DNA of the species in which the screen is conducted. Therefore, we tested several methods (sonication and digestion with DNAse and MNase) for controlled fragmentation of genomic DNA. Ultrasonic degradation proved to be inadequate to obtain small (~20 bp) DNA fragments and DNAse digestion was poorly controllable (Additional file 2: Figure S1A). By comparison, 7.5 Units of micrococcal nuclease (MNase), a commonly used prokaryotic enzyme with minimal cleavage preferences reproducibly digested 1 μg of genomic DNA into 10–200 bp fragments when incubated at 37 °C for 15 min (Fig. 2a). This enabled us to obtain DNA fragments of the desired size (between 20 and 30 bp) by size separation and extraction from 20 % PAGE gels (Fig. 2b). Following gel excision, fragments were recovered from the gel using the Crush-and-Soak method [34]. Subsequent purification by phenol-chloroform and repair of 5′ and 3′ ends reproducibly yielded around 2 % of the starting amount, which for the following experiments was equivalent to approximately 300 ng, representing approximately a 100000-fold coverage of a typical mammalian genome.

Generation of high complexity lentiviral gRNA pools relies mainly on efficient cloning of targeting sequences into an expression vector. Therefore, we initially modified the 2^nd^ generation lentiviral expression vector PLKO.1 and included, instead of the commonly used shRNA expression cassette, the U6 promoter, the gRNA stem loop and terminator sequences (from plasmid MLM3636) and refer to hereafter as “gRNA-PLKO1” (Fig. 1b). This construct enables the efficient cloning of any chosen gRNA targeting sequence downstream of the promoter and upstream of the gRNA stem loop using Gibson assembly [35]. To enable efficient assembly of complex pools of genomic DNA fragments into gRNA-PLKO1, linker sequences providing homology to the plasmid were ligated to the fragmented genomic DNA (Fig. 1b). For this step, 5′- and 3′-linker fragments were amplified from gRNA-PLKO.1 by PCR. Amplified linkers were digested with restriction enzymes cutting at the external ends of the linkers to provide directionality to the blunt end ligation reaction, which adds one linker to each repaired end of the digested double stranded genomic DNA fragment (Additional file 2: Figure S1B). Due to lack of 5′phosphate groups at the linker amplicons linker-to-linker ligation does not occur. Given that only fragmented genomic DNA provides 5′phosphates for ligation, a nick translation step was necessary to ‘seal’ the ligation products. After PCR, fragments containing both linkers were size-selected and inserted into the gRNA vector gRNA-PLKO.1 via Gibson assembly (Fig. 1b). To investigate the reproducibility of the CORALINA protocol for different cloning strategies and the impact of different overhang sequences on the efficiency of library assembly we used three primer pairs yielding differently sized amplicons L1, L2 and L3 (Fig. 1 and Materials and Methods). The three amplicons were used in 16 Gibson assembly reactions each to incorporate human and mouse genomic DNA into the lentiviral gRNA expression vector efficiently. Following a reaction cleanup, the purified Gibson assembly reactions were electroporated into freshly prepared highly competent bacteria (>10^10 Colony-forming units/ ug DNA) from which the plasmid library was extracted (see Materials and Methods).

The scale and consistency of the generated gRNA libraries generated was analyzed individually for the human L1, L2 and L3 libraries and as pools for the three (L1-L3) mouse libraries through next generation sequencing (Fig. 1a). The high expected complexity of CORALINA libraries made a complete description of all contained gRNA sequences an intricate matter, since read numbers required for sufficient coverage can only be obtained at vast expenses. Instead, we aimed to produce representative information from each cloned library through sequencing of a gRNA subset (up to 10^6 reads). This proved not only to be sufficient to determine the efficiency and reproducibility of the method and to analyse structural features of the generated gRNAs, it also enabled us to investigate the distribution, the genomic categories and the specificity of gRNA targeting sites and even allowed us a rough estimation of gRNA numbers in the original library pool (see below).

**Fig. 3:**
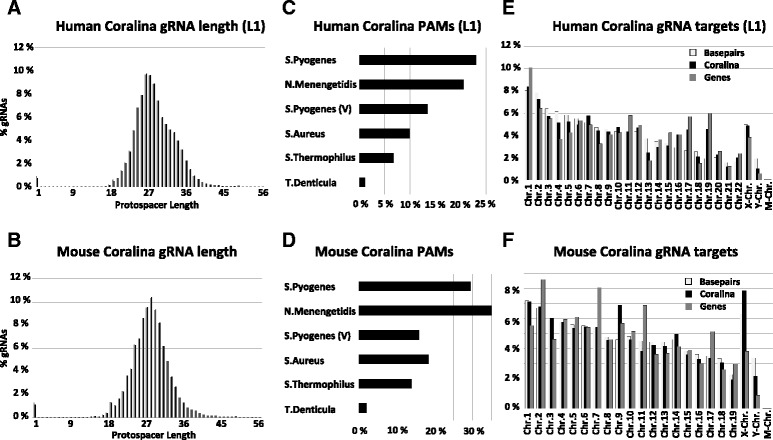
Analysis of CORALINA gRNAs. Next generation sequencing of CORALINA libraries have been used to analyze generated libraries. **a**, **b** Quantification of protospacer length of human (**a**, L1) and mouse (**b**, “pooled”) gRNA libraries. Protospacers are mostly between 18 and 36 bp long. Shown are protospacer lengths detected by forward (*black*) and reverse (*grey*) sequencing reads. **c**, **d** Proportions of human (**c**) and mouse (**d**) CORALINA gRNA protospacer aligning to genomic sites containing functional PAMs of published Cas9 proteins. **e**, **f** Distribution of sequenced gRNA protospacer alignments to the human (**e**) or mouse (**f**) genome. While the number of associated CORALINA gRNAs (*black bars*) is overall correlating to the size of the respective human chromosome (*white bars*), GC- and gene-rich chromosomes (*grey bars*, 16, 17, 19) are overrepresented relative to their size

**Fig. 4:**
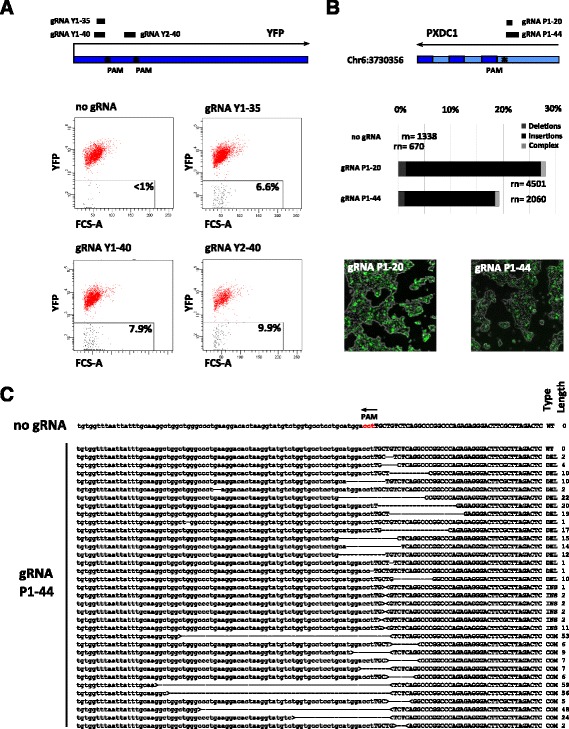
Functional analysis of CORALINA gRNAs. **a** *Top*: Overview of gRNAs used in experiments to target YFP. gRNA Y1-35, gRNA Y1- 40, gRNA Y2-40 target YFP and contain protospacer with 35 or 40 bp length respectively. Bottom: Flow cytometry reveals that gRNAs with long protospacer (35 or 40 bp) efficiently target the Cas9 protein and induce mutations (detected through YFP loss, transfection rate ca. 30 %). **b** *Top*: Schematic depicting a CORALINA derived gRNA targeting the third last intron of the human gene PXDC1 (gRNA P1-44, 44 bp protospacer). gRNA P1-20 is trimmed from the 5′ end to yield a 20 bp protospacer. Below: Bargraph depicting percentage of NGS reads displaying indels resulting from coexpression of wild-type Cas9 with the two gRNAs in HEK293T cells. While NGS sequencing reads of control cells reveal only wildtype sequences, reads derived from cells transfected with gRNA P1-20 or gRNA P1-44 (and S. Pyogenes Cas9) displayed genetic alteration around the targeting sites. Bottom: Microscopy images indicating transfection efficiency is ca. 30 %. **c** List of the most frequently sequenced alterations generated with gRNA P1-44 and classified as WT (wild-type), INS (insertion), DEL (deletion) or COM (complex). Complex cases relate to cases where more than one insertion or deletion has happened. The sequence highlighted with capital letters correspond to the target of the guide RNA. For insertions and complex events, the ‘>’ ‘<‘mark the location of the event. See Additional file 4: Figure S4A for alterations induced by gRNA P1-20

First, obtained sequencing reads were analyzed for gRNA protospacer sequences, the only variable part within the vector library; next, we determined their lengths. The lengths of protospacers are critical, since short gRNAs (e.g. with less than 18 bp protospacer), although still potentially functional in targeting Cas9 to genomic DNA, are on average less likely to possess single genomic targeting sites. Figure 3 shows that a vast majority of CORALINA gRNAs contains protospacer of 18 bp or longer. Each of the generated CORALINA libraries possessed an average protospacer length of 26–29 bp and only a low proportion of vectors lacking functional protospacers at all, indicating high reproducibility of the method and only a moderate effect of the used linker sequences on the efficiency of the method (Fig. 3 a, b, Additional file 3: Figure S2). Since a proportion of CORALINA gRNAs contained protospacers longer than 30 bps, we examined whether these elongated gRNAs would still be able to guide Cas9 to genomic targeting sites. We tested this using two independent assays based on flow cytometry and next-generation sequencing, respectively (Fig. 4a and b). By employing neural stem cells constitutively expressing the YFP transgene, we show, that designed gRNAs targeting YFP, but with protospacer lengths of 35 or 40 bp (gRNA Y1-35, gRNA Y1-40, gRNA Y2-40) efficiently induce functional knockouts of YFP when combined with S.pyogenes Cas9 (Fig. 4a).

We next assayed five randomly selected elongated gRNAs mapping to unique genomic sites followed by NGG PAMs from the human CORALINA libraries (L1). These gRNAs possessed targeting sites inside or close to the genes *PXDC1* (gRNA P1-44), *HS3ST3B1* (gRNA H1-46), *PCDH8* (gRNA P2-40), *ZNF790* (gRNA Z1-35) and *PIK3AP1* (gRNA P3-35) respectively. As shown in Fig. 4b, c and Additional file 4: Figure S4 A-E these gRNAs (containing protospacer of 35 to 46 bp in length) are able to induce targeted mutagenesis when co-expressed with *S.pyogenes* Cas9 in human cells. The mutations were structurally similar to those produced using shortened versions of the same gRNAs containing 20 bp protospacer (e.g. gRNA P1-20) resulting in the three types of CRISPR indels (deletions, insertions and complex mutations, Fig. 4b, c and Additional file 4: Figure S4).

Subsequently, we used the information of the sequenced library reads to obtain a rough estimate of library complexity. For this we utilized the frequency of single protospacer sequences obtained during the NGS run. The estimate is based on the following rationale: The lower the complexity of the underlying CORALINA library, the more often individual protospacer should be sequenced more than once. However, obtained sequenced samples of each of the four CORALINA libraries contained mainly “singular” sequences (ca. 80–90 %, Additional file 5: Table S1) that were only represented by a single read in the sequencing experiment. Moreover, sequenced samples of the three human CORALINA libraries analyzed by NGS also have very few sequences in common (Fig. 1c). Both of this indicated that CORALINA libraries exhibit a far higher number of different protospacer sequences than sequencing reads obtained (ca.10^6) and consequently likely represent a considerable proportion of the genome. To confirm this finding we extrapolated library complexity from the sequencing data using a Bayesian model ([38] see Material and Methods)), which suggests a library size of 5×10^7–10^9 individual gRNA sequences.

Next, we used Bowtie [37] to align the obtained gRNA protospacer sequences to the mouse and human reference genomes and to determine their genomic distribution. To predict how useful each of the generated CORALINA libraries would be in conjunction with the different published classes of Cas9 variants, we determined the proportion of gRNA sequences possessing genomic targeting sites followed by a functional PAM. The libraries presented here contain scaffolds specific to S. Pyogenes Cas9 proteins and variants (for which up to 40 % of targeting sites could be functional, containing PAMs with ‘NAG’, ‘NGG’, ‘NGA’ and ‘NGCG’ [4, 39] and ca. 23–30 % contain a canonical *S. Pyogenes* PAM (‘NAG’, ‘NGG’)). As depicted in Fig. 3 c, d (and Additional file 3: Figure S2), a majority of individual gRNA sequences of both, mouse and human CORALINA sequences would with minor adjustments of the vectors also be adaptable to other published Cas9 variants [40, 41]. Moreover, the proportions of individual PAM sequences are similar between human and mouse CORALINA sequences and almost identical among the three human libraries indicating reproducibility both, among and between species (Additional file 3: Figure S2).

**Fig. 5:**
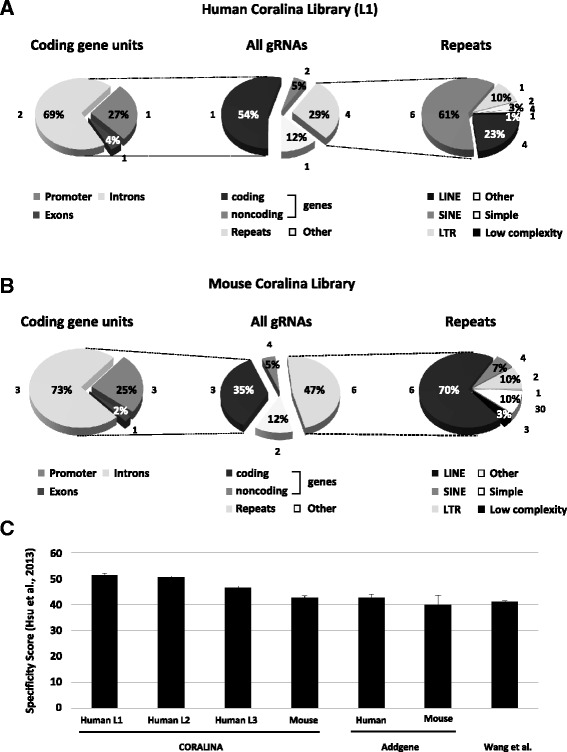
Analysis of CORALINA libraries. Bowtie has been used to classify targeting sites of gRNAs derived from human (L1, **a**) or mouse (“pooled”, **b**) CORALINA libraries. Pie charts indicating relative proportion of functional domains bound by gRNAs (*middle*). gRNA protospacer aligning to coding gene units (*left*) or repeats (*right*) are further sub-classified. Promoters are defined here as genomic sequences between 10 kb upstream to the transcriptional start site. Coding and noncoding gene and repeat information has been derived from UCSC. Numbers next to the sectors depict the median number of genomic alignments for the selected group of gRNAs. **c** Depiction of average specificity score of CORALINA libraries calculated according to [45] and compared to available gRNAs (Addgene) and a published gRNA library [49]. *Error bars* depict the standard error of the mean

**Fig. 6:**
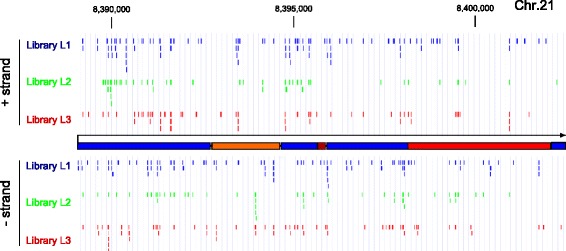
Visual representation of protospacer alignments to a ribosomal gene unit. Shown are sequences detected in the sequencing reads of three human CORALINA libraries (L1, L2, L3) which correspond to a ribosomal gene unit on chromosome 21. Alignments to the positive DNA strands are shown above, those to the negative DNA strand below the gene structure using the UCSC genome browser (*blue from left to right*: 5′ETS, ITSs, 3′ETS; *orange*: 18S rRNA, 5.8S rRNA, 28S rRNA)

To quantify the distribution of gRNA targeting sites we mapped the obtained sequences to the human and mouse genomes. Figure 3e and f shows that gRNA protospacers are derived from each chromosome including gonosomes and the mitochondrial genome. The chromosomal distribution of gRNA targeting sites is almost identical for the three human CORALINA libraries (Fig. 3, Additional file 3: Figure S2) despite the lack of shared protospacer sequences in the analyzed NGS samples, indicating a high reproducibility of the method (Fig. 1c). While the numbers of individual gRNA targeting sites are generally correlating to the size of the chromosomes, it is also apparent that chromosomes overrepresented in gRNA numbers in all three human CORALINA libraries are those harboring a particular high GC content (and also a high relative amount of genes, e.g. human Chr. 16, 17 and 19, but not gene-poor Chr. 13 and 18). This effect is less pronounced in the CORALINA library generated from the relatively GC-rich mouse genome. This slight genomic skew and the elevated average GC content of the gRNA libraries (48–62 %, Additional file 5: Table S1) might be derived from a cleavage preference of Micrococcal nuclease, since the AT content 5′ and 3′ to the cloned cutting sites appear increased [42] (Additional file 6: Figure S5). To rule out a functional bias in the CORALINA method we grouped the mapped sequences according to annotation categories (Fig. 5 and Additional file 7: Figure S3). Analysis reveals that a large proportion of the CORALINA gRNAs possesses targeting sites inside genes (promoter, exons and introns: 59 % (human L1), 40 % (mouse); as a comprehensive example for their distribution on a complex gene unit, see Fig. 6). Most genes associated with protospacer are encoding proteins (coding transcription units: 55 % (human L1), 35 % (mouse); noncoding transcription units: 5 % (human L1), 5 % (mouse), Fig. 5). gRNAs specific for multi-copy domains (like transposons, retroviral, simple, tandem or interspersed repeats) are present in the generated libraries, but relative to their genomic distribution underrepresented (human 29 %, mouse 47 %, Fig. 5). Differences between the human and mouse libraries, specifically in the distribution of protospacers representing particular repeat classes, can likely be explained by their different genomic composition [43, 44]. Importantly, despite the lack of shared protospacer sequences in the analyzed NGS samples of the three human CORALINA libraries (Fig. 1c), genomic categories are almost equally represented among the gRNA targeting sites, indicating high reproducibility and robustness of the method (Multinomial equivalence test, confidence value 0.99, Material and Methods). To investigate the specificity of the obtained gRNA sequences we determined for each of them the number of potential targeting sites in the appropriate genome. As expected, gRNA protospacer associated with repeat sequences (simple, low complexity, LTR, SINE or LINEs) often contain several genomic binding sites (represented by a relative high median of target sites, human L1: 4, mouse: 6). In contrast, gRNA sequences targeting human coding genes possess often a single genomic targeting site (represented by a median of 1, Fig. 5). While this is especially predominant in exonic sequences, even gRNAs targeting promoters (median target sites: 1), introns (2), noncoding gene units (2) and non-annotated regions (other, 1) have often unique targeting sites (Fig. 5). This relative high specificity of CORALINA gRNAs (even comparable to published gRNAs or synthetized gRNA libraries) has been independently validated by determination of established *in silico* quality scores (Fig. 5c, [45]). Thus, excellent coverage and satisfactory specificity indicate a high potential for CORALINA libraries in a large number of approaches.

## Discussion

We present here a new, simple, time- and cost-effective method to generate high complexity gRNA libraries from any source of DNA (CORALINA). The presented method is not only applicable to uncommon model systems; it also does not depend on the availability of reliable sequence information. As a proof of principle, we generated CORALINA libraries from two large mammalian genomes, Mus musculus and Homo sapiens, determined the obtained sequences through next generation sequencing and used cross species validation to confirm its reproducibility. Supported by a recent publication elegantly constructing gRNAs from a prokaryotic genome (E. Coli) [29] we show that employing nucleases is a highly efficient approach for gRNA library generation (CRISPR EATING). In both approaches only a minority of gRNAs contain a PAM for a specific Cas9 variant when libraries are generated. In contrast to CRISPR EATING, however, CORALINA libraries have the potential to contain more possible gRNA sequences for one single Cas9 variant (e.g. S.Pyogenes, Additional file 6: Figure S5). Bioinformatic and statistical analysis revealed that CORALINA yields high complexity libraries (5×10^7–10^9) comprehensively covering the genome. The obtained protospacers represent all genomic classes including mitochondrial, ribosomal, regulatory, coding and noncoding transcription units. Screening with CORALINA will allow the revelation of functional hits from both, single copy regions and classes of multicopy sequences (the value of which recently has been impressively demonstrated [46]), through the versatility of the generated libraries.

While CORALINA allows the efficient and cost-effective cloning of virtually all functional gRNA sequences from a specific genome, those also contain gRNA sequences, which, due to the lack of PAM sequences, are not able to guide Cas9 proteins to their targeting sites. While this specific shortcoming is a direct consequence of the high complexity of CORALINA libraries, its negative impact can likely be diminished. Since gRNAs lacking PAMs are not biologically active, increased cell number or gRNA number per cell (e.g. through higher MOI) would not necessarily interfere with screening. Since PAMs are easy to determine in silico, those gRNAs without could even serve as negative controls helping to determine thresholds for candidate hits and make ‘spiked in’ negative controls obsolete. Furthermore, since several laboratories currently work on Cas9 variants being dependent on new PAM sequences (or none at all) the momentary disadvantage of CORALINA libraries could eventually turn into an advantage [39–41, 47]. Indeed, CORALINA gRNAs possess targeting sites containing all PAM sequences published to date making this method in principle applicable for each Cas9 variant and even combinatorial use. As part of the bioinformatics analysis, protospacer lengths of CORALINA gRNAs were analysed as well. Conventionally designed gRNAs usually contain a protospacer of 20 bp, since shorter protospacer have often multiple on-target sites (and thus are less specific). CORALINA generates gRNAs with relatively long targeting sequences (between 18 and 40 bps, Fig. 3), which, as shown here, are functional in targeting the CRISPR complex (Fig. 4 and Additional file 4: Figure S4).

Each screening approach is different; some experimental setups might not be adaptable to large scale approaches and few might allow screening a mammalian genome to saturation. However, for some approaches (e.g. those with low false positive rates) CORALINA could allow for the first time the discovery of functional hits without an intense bias to few targeting sites and ORFs. Moreover, we envision that using multiple rounds of reiterative screening would make CORALINA applicable to many positive selection screens. If after each round of screening, gRNA constructs are extracted from selected cells and used as templates for the generation of subsequent gRNA libraries, true positive hits would continuously propagate while library complexity decreases with every round. Since CORALINA can in principle be applied to any source of DNA, the achieved library complexity decreases with the complexity of the input DNA. While the genomes used here for proof of principle experiments belong to the largest ones studied, most model organisms possess considerably smaller genomes. The libraries generated here contained all genomic classes comprehensively (including coding and non-coding transcripts, exons, introns, gene promoters, mitochondrial sequences and other features) indicating that genomic subsets (ChIPed DNA, mtDNA, reduced representation DNA) would be a suitable input for targeted CORALINA libraries in the future.

## Conclusions

The development of CORALINA makes it possible to generate pools of virtually genome-wide gRNA libraries at low cost. This allows not only conducting functional screens at an unmatched genomic depth, but simultaneously makes approaches possible that have been so far impractical. CORALINA enables first of all a simple generation of large scale libraries from any source of DNA (including species lacking reliable sequence information). Secondly, CORALINA allows genetic screens for functional non-coding transcripts and elements, a highly promising approach so far only practical on individual genomic elements [32]. Third, through the use of enzymatic inactive Cas9 variants shuttling chromatin modifying enzymes to defined genomic sites, CORALINA might also allow the implementation of genome-wide epigenetic screens. Since a large proportion of the genome is suspected to play a regulatory role [48] these approaches inherently depend on genuine genome-wide libraries.

## Additional files

Additional file 1: Supplementary methods. Supplementary information to bioinformatic and statistical analysis, primer sequences and supplementary methods. (PDF 276 kb)
Additional file 2: Figure S1. (A) DNAse digestion (left) or sonication (right) has proven inappropriate for the controlled and efficient generation small (<30 bp) DNA fragments from genomic DNA. (B) Overhang adapters enabling Gibson assembly have been generated using PCR (left side) and cut (right side) to produce asymmetric linker ends (see Fig. 1). (PDF 2811 kb)
Additional file 3: Figure S2. Analysis of CORALINA gRNAs. Next generation sequencing of CORALINA libraries have been used to analyze generated libraries. (A, B) Quantification of protospacer length of human L2 (A, C, E) and L3 (B, D, F) gRNA libraries. Protospacers are mostly between 18 and 36 bp long. Shown are protospacer lengths detected by forward (black) and reverse (grey) sequencing reads. (C, D) Proportions of CORALINA gRNAs aligning to genomic sites containing functional PAMs of published Cas9 proteins. (E, F) Distribution of sequenced gRNA protospacer alignments to the human genome. While the number of associated CORALINA gRNAs (black bars) is overall correlating to the size of the respective chromosome (white bars), GC- and gene-rich chromosomes (grey bars, 16, 17, 19) are overrepresented relative to their size. (PDF 70 kb)
Additional file 4: Figure S4. Functional analysis of CORALINA gRNAs. (A) List of most frequently sequenced alteration generated with gRNA P1-20. (B-E) Top: Schematic depicting CORALINA-derived gRNAs targeting regions in or near various human genes (HS3ST3B1 gRNA H1-46, 46 bp protospacer, PCDH8, gRNA P2- 40, 40 bp protospacer, ZNF790, gRNA Z1-35, 35 bp protospacer, PIK3AP1, gRNA P3-35, 35 bp protospacer). Control gRNAs have been shortened from the 5′ end to yield a 20 bp protospacer. Right: Bargraph depicting percentage of NGS reads displaying indels after targeting wild-type Cas9 using CORALINA-derived gRNAs in HEK293T cells. Below: List of the most frequently sequenced alterations generated by CORALINA and control gRNAs. (PDF 320 kb)
Additional file 5: Table S1. Quantification of NGS read number and GC content for the four analysed NGS sequencing samples. (PDF 19 kb)
Additional file 6: Figure S5. Comparison of CORALINA libraries to an alternative method used for the generation of large-scale gRNA libraries targeting the E.coli genome (CRISPR-EATING) [29]. (A) Theoretical number of E.coli gRNAs with S.Pyogenes PAM sequences accessible to CRISPR-EATING and CORALINA respectively. (B) GC content of the different gRNA libraries and of the genomes they were generated from. (C) Nucleotide frequency at the 5′ and 3′ ends of the gRNAs incorporated into libraries corresponding to the cutting sites of the nucleases/restriction enzymes used. (PDF 40 kb)
Additional file 7: Figure S3. Analysis of CORALINA libraries. Bowtie has been used to classify targeting sites of gRNAs derived from human (L2, A or L3, B) CORALINA libraries. Pie charts indicating relative proportion of functional domains bound by gRNAs (middle). gRNA protospacer aligning to coding gene units (left) or repeats (right) are further sub-classified. Promoters are defined here as genomic sequences 10 kb upstream the transcriptional start site. Coding and noncoding gene and repeat information has been derived from UCSC. Numbers next to the sectors depict the median number of genomic alignments for the selected group of gRNAs. (PDF 73 kb)

## Abbreviations

Cas9: CRISPR-associated protein 9
ccfDNA: Circulating cell free DNA
ChIP: Chromatin immunoprecipitation
CORALINA: Comprehensive gRNA library generation through controlled nuclease activity
CRISPR: Clustered regularly interspaced short palindromic repeats
ctDNA: Circulating tumor DNA
gRNAs: Engineered guide RNA molecules
MOI: Multiplicity of infection
mtDNA: Mitochondrial DNA
PAM: Protospacer adjacent motive
