# Supplementary material for: CORALINA: a universal method for the generation of gRNA libraries for CRISPR-based screening
Source: BMC Genomics. 2016 Nov 14;17:917. doi: 10.1186/s12864-016-3268-z (PMC5109649; doi:10.1186/s12864-016-3268-z)
Supplement: Additional file 1: — Supplementary methods. Supplementary information to bioinformatic and statistical analysis, primer sequences and supplementary methods. (PDF 276 kb) [file 12864_2016_3268_MOESM1_ESM.pdf]

Supplementary methods:

CORALINA Library QC by sequencing

A fragment 113 bp in length and comprising the gRNA targeting sequence was amplified from the library (100 ng input) using Long Amp Taq 2X Master Mix (NEB) and 1µl of each primer gRNA-Upstream-50-F (10 µM, 50-AAGTATTTCGATTTCTTGGCTTTATATATCT) and gRNA-Downstream-19-R (10 µM, 50-CGGACTAGCCTTATTTTAACTTGC) in a total volume of 25 µl. Cycling conditions were 1 cycle at 94˚C for 30 s, 10 cycles of 94˚C for 30 s, 52˚C for 30 s, 65˚C for 15 s and final elongation at 65˚C for 10 min. The reaction was purified using Agencourt AMPure XP (Beckman Coulter) with a sample-to-bead ratio of 1:2. Ends were phosphorylated using T4 polynucleotide kinase (NEB). Reactions were set up as follows: 5 µl T4 DNA ligase buffer including ATP, 2 µl T4 PNK (10 U/µl) in a total reaction volume of 50 µl. Reactions were bead-purified using a sample-to-bead ratio of 1:2. Pre-annealed Mis-Seq adapters (Adapter-InPE-1.0 5'- [Phos]GATCGGAAGAGCACACGTCT, Adapter-InPE-2.0 5'- ACACTCTTTCCCTACACGACGCTCTTCCGATC*T) were ligated to the ends of the PCR amplicon using the Quick ligation kit (NEB) with an adapter-to-PCR-insert ratio of 10:1 using 5 µl ligase enzyme and incubating at 18˚C for 2 h. This was followed by bead cleanup and nick translation using Long Amp Taq 2X Master Mix (NEB) in a total volume of 50 µl incubated at 72˚C for 20 min, followed by an additional bead cleanup. PCR amplification with KAPA HiFi PCR kit (Kapa Biosystems) with indexed reverse primers was used for indexing the sequencing libraries. Amplification conditions were as follows: 10 µl Kappa GC buffer, 1.5 µl 10 mM each dNTP mix (NEB), 2 µl 25 mM primer InPE-1.0-F (5'-

AATGATACGGCGACCACCGAGATCTACACTCTTTCCCTACACGACGCTCTTCCGATC*T) and 2 µl 25 mM of indexed reverse primer (list of primers is attached below), 2.5 µl DMSO, 1 µl KAPA HiFi polymerase in a total of 50 µl. Cycling conditions were 1 cycle at 95˚C for 2 min, 6 cycles of 98˚C for 20 s, 60˚C for 15 s, 72˚C for 15 s and 1 cycle at 72˚C for 5 min. Indexed reverse primer sequences were as follows:

Name	Sequence
InPE-2.1-R	CAAGCAGAAGACGGCATACGAGATCGTGATGTGACTGGAGTTCAGACGTGTGCTCTTCCGATC*T
InPE-2.2-R	CAAGCAGAAGACGGCATACGAGATACATCGGTGACTGGAGTTCAGACGTGTGCTCTTCCGATC*T
InPE-2.3-R	CAAGCAGAAGACGGCATACGAGATGCCTAAGTGACTGGAGTTCAGACGTGTGCTCTTCCGATC*T
InPE-2.4-R	CAAGCAGAAGACGGCATACGAGATTGGTCAGTGACTGGAGTTCAGACGTGTGCTCTTCCGATC*T

Reactions were cleaned up using Agencourt AMPure XP (Beckman Coulter) according to the manufacturer's instructions and quantified using the Qubit dsDNA BR Assay (Life Technologies). A 4 nM library including a 5 % spike-in of PhiX control DNA was prepared for sequencing on the Illumina MiSeq according to manufacturer's instructions.

Bioinformatics analysis of the gRNA libraries

The raw-reads of eight FASTQ files (forward and reverse reads of the three human L1, L2 and L3 libraries and the pooled (L1-L3) mouse library) were trimmed for the illumina adapters, U6 promoter sequences (TGTGGAAAGGACGAAACACCG) and the gRNA hairpin (GTTTTAGAGCTAGAAATAGCAAGTTAAAATAAGGCTAGTCCG) considering both read directions using Cutadapt (https://cutadapt.readthedocs.org/en/stable/) and the FASTX toolkit

(http://hannonlab.cshl.edu/fastx_toolkit/). Custom-designed python scripts were used for these and all subsequent steps, for instance to determine the sequence number and protospacer length. The Code is available at hmgubox (https://hmgubox.helmholtz-muenchen.de:8001/d/6c6e75236e/; password: Coralina).

Next, unique gRNA protospacer sequences larger than 17bp were used to analyze sequencing-sample overlaps (Venn diagram was generated using Euler APE (Micallef and Rodgers, 2014)) and to map protospacers to the human reference genome hg19 or the mouse reference genome mm10 obtained from UCSC (http://genome.ucsc.edu/) using Bowtie (version 1.1.2) allowing no mismatches [36]. To investigate the occurrences of PAM sequences next to the gRNA binding sites the following published PAM sequences were used for the analysis: S.Aureus, NNGRR, [37], S.Pyogenes, NAG, NGG [4], S.Pyogenes variants NGCG, NGAT, NGAG, NGAC, NGAA [38], N.Menengitidis, NNNNGANN, NNNNGTTN, NNNNGNNT [39], T.denticola, NAAAAN [39], S.termophilus, NNAGAA, NNGGAA, NNAGGA, NNAGCA, NNACCA, NNATAA, NNAAAA, NNGGGA [38]. In case protospacers had multiple perfect genomic targeting sites up to ten were considered. PAM sequences derived from one Cas9 class were grouped together and counted at most once. For the genomic analysis known genes and repeats of the human (hg19) and mouse (mm10) reference genomes were downloaded from UCSC. Gene positions have been expanded for 10kb upstream sequence to take promoter sequences into account. For each generated alignment it was examined sequentially, whether they are located on coding genes, non-coding genes, repeats or none of these categories. In case gRNA protospacers had multiple perfect genomic targeting sites one site was randomly chosen. For

gRNA alignments located on coding genes it was sequentially determined whether they correspond to an exon, a promoter (defined as up to 10kb upstream of the TSS) or an intron region. gRNA alignments located on repeats were further analyzed sequentially to determine repeat subclasses: Simple repeats, Low complexity, LTR, LINE, SINE and Others. In all functional genomic analyses, each gRNA was counted only in the first genomic class detected, meaning that a read mapping to one genomic region (e.g. coding gene) was not counted on another region (e.g. non-coding gene), even if more allocations existed. To compute the median of targeting sites all possible occurrences of each protospacer were assessed.

To investigate hits covering the DNA sequence of a representative ribosomal gene unit (rDNA), the corresponding 13.5 kilobase sequence on Chromosome 21 (chr21: 8389035 – 8402378) was extracted from the UCSC website (http://genome.ucsc.edu/). Bowtie version 1.1.2 was then used to index the rDNA and align all protospacers longer than 17bp obtained from the sequencing of the three human libraries respectively. No mismatches were allowed in the alignment. Protospacers that were found to align to the indexed rDNA were subsequently divided into hits on the + and the – strand of the DNA, and the UCSC website was then used to illustrate their distribution across the ribosomal gene.

To estimate the complexity of the original pool of gRNAs from the sequenced samples of CORALINA libraries, we assumed the sequenced samples to be a multinomial draw (sampling with replacement) from the original pool of gRNAs. In a Bayesian approach, we derived the posterior density for the number of different gRNAs in the original pool. We sampled from the

posterior distribution using the Markov chain Monte Carlo algorithm [40]. Details of the statistical model are as follows:

By S_i, $i = 1, \ldots, l$, we denote the *different* sequences in the original pool. Each S_i is contained in that pool $a \cdot c_i$ times, where a stems from the number of amplifications and c_i is the number of occurrences of S_i in the genome. Only $k \leq l$ of the sequences are contained in the sequenced pool. Without loss of generality, let $i = 1, \ldots, k$ be the indices of actually read sequences. As a is a very large number, we can consider the read sequences to be a random draw with replacement from $\{S_1, \ldots, S_l\}$ with weights $c_1, \ldots, c_l$. Let $x_1, \ldots, x_l$ be the observed frequencies of $S_1, \ldots, S_l$ in the sequenced pool. Due to the above assumption, we have $x_{k+1} = \cdots = x_l = 0$ if $l > k$. The statistical model is

$$(x_1, \ldots, x_l) \sim \text{Multinomial}(m, (q_1, \ldots, q_l))$$

with $m = \sum_{i=1}^{l} x_i$ and $q_i = a_i / \sum_{j=1}^{l} a_j$ for all i. The likelihood function of the number l of different sequences in the original pool reads

$$L(l; x_1, \ldots, x_l) = \binom{m}{x_1, \ldots, x_l} \prod_{i=1}^{l} q_i^{x_i} = m! \prod_{i=1}^{k} \frac{q_i^{x_i}}{x_i!}$$

From the above model one would expect to see a positive correlation between x_1 and c_1. In our data, this is however not the case, indicating that l is several magnitudes larger than k. In the following, we restrict our analysis to those reads that occur only once in the genome, i.e. $c_i = 1$. It then follows that $q_i = 1/l$ for all i.

Maximization of the likelihood function yields the maximum likelihood estimate $\hat{l} = k$, i.e. the number of different sequences in the original pool is estimated to equal the number of different observed reads. While this is statistically correct, it is biologically implausible. We

hence decided to use a Bayesian approach for parameter estimation which does not only take into account the information from the observations, represented by the likelihood function, but also external biological knowledge, expressed through a prior density. These two components result in the posterior density of l:

$$\text{post}(l|x_1, \dots, x_k) = \frac{L(l; x_1, \dots, x_k)\text{prior}(l)}{\sum_{l'=k}^{\infty} L(l'; x_1, \dots, x_k)\text{prior}(l')}$$

for all $l \geq k$. Calculation of the denominator is problematic for numerical and computational reasons. We hence employed the Metropolis-Hastings algorithm, which belongs to the class of Markov chain Monte Carlo (MCMC) methods, for posterior estimation of l [1]. This technique does not require calculation of the denominator.

The prior density represents our expectation about the parameter l without taking into account the data $x_1, \dots, x_k$. We achieved good mixing of the MCMC algorithm for Gaussian prior densities with mean values between $5 \cdot 10^7$ and 10^9 and standard deviation 10^5. For uniform prior densities or Gaussian prior densities with lower mean values we did not observe convergence of the algorithm. For each MCMC run, we performed at least 10^8 iterations. In case of good mixing, the burn-in period was never longer than 10^5. As expected, the posterior distributions strongly depended on the prior distributions. Posterior means were of the same magnitude as prior means. The fact that good convergence was achieved only for prior means larger than $5 \cdot 10^7$ leads us to the conclusion that a number of different gRNAs in the original pool between $5 \cdot 10^7$ and 10^9 is in conformity with the observed data. All calculations were carried out in R.

To test whether equality of frequencies between the three human NGS samples can be assumed within certain error margins, an equivalence test was employed which, in contrast to the more common significance tests, provide evidence for similarities rather than differences. In particular, we used a multinomial equivalence test by Wellek [41] to test whether each of the datasets stems from a reference multinomial distribution with the vector of probabilities for the single categories being derived from the union of all datasets (tolerance level 0.02, confidence level 0.99).

Comparison with CRISPR-EATING libraries

CRISPR EATING NGS data was extracted from [7]. Raw reads were trimmed to extract the gRNA sequences and aligned to the E.coli genome (strain K12, substr. MG1655) using Bowtie. The GC content was calculated as described before. The Code is available at hgmubox (https://hmgubox.helmholtz-muenchen.de:8001/d/6c6e75236e/; password: Coralina).

Calculation of gRNAs specificity scores

For each set of gRNAs, the average specificity score was calculated from individual specificity scores generated using the Benchling online tool [2]. For this purpose, 1000 CORALINA gRNAs containing a NGG PAM were randomly selected and (if longer) cropped to 20bp at the 5’ (not influencing the specificity score). 1000 gRNAs randomly selected from a published library [3] and all human and mouse gRNAs currently available on Addgene were also scored for comparison.

Functional validation of gRNAs longer than 30 bp from the CORALINA library

gRNAs were selected from the sequencing output of the human L1 sample of the CORALINA library that were 35, 40, 44 or 46 bp in length, align uniquely to the genome and are followed by an NGG PAM sequence. These gRNA sequences were cloned into px458 (Addgene plasmid 48138), from which a gRNA can be co-expressed with a wild-type Cas9-T2A-GFP fusion protein. The vector backbone was digested with BbsI (NEB), the primers pre-annealed and inserted into the backbone by Gibson cloning.

Name	Sequence
P3-20bp-chr10-98499080-2F	TTGTGGAAAGGACGAAACACCGCTCCCAAAGTGCTGGGATCGTTTTAGAGCTAGAAATAGCAA
P3-20bp-chr10-98499080-2R	TTGCTATTTCTAGCTCTAAAACGATCCCAGCACTTTGGGAGCGGTGTTTCGTCCTTTCCACAA
P3-35bp-chr10-98499080-1F	TTGTGGAAAGGACGAAACACCGTCCACCTGCCTCAGCCTCCCAAAGTGCTGGGATCGTTTTAGAGCTAGAAATAGCAA
P3-35bp-chr10-98499080-1R	TTGCTATTTCTAGCTCTAAAACGATCCCAGCACTTTGGGAGGCTGAGGCAGGTGGACGGTGTTTCGTCCTTTCCACAA
Z1-20bp-chr19-37340687-2F	TTGTGGAAAGGACGAAACACCGGGGGGGGCAAGATCGTGTAGTTTTAGAGCTAGAAATAGCAA
Z1-20bp-chr19-37340687-2R	TTGCTATTTCTAGCTCTAAAACTACACGATCTTGCCCCCCCCGGTGTTTCGTCCTTTCCACAA
Z1-35bp-chr19-37340687-1F	TTGTGGAAAGGACGAAACACCGGAGTGTGTGGAGGTGGGGGGGGCAAGATCGTGTAGTTTTAGAGCTAGAAATAGCAA
Z1-35bp-chr19-37340687-1R	TTGCTATTTCTAGCTCTAAAACTACACGATCTTGCCCCCCCCACCTCCACACACTCCGGTGTTTCGTCCTTTCCACAA
P2-20bp-chr13-53435625-2F	TTGTGGAAAGGACGAAACACCGTGCAGTGAGCTGAGATCACGTTTTAGAGCTAGAAATAGCAA
P2-20bp-chr13-53435625-2R	TTGCTATTTCTAGCTCTAAAACGTGATCTCAGCTCACTGCACGGTGTTTCGTCCTTTCCACAA
P2-40bp-chr13-53435625-1F	TTGTGGAAAGGACGAAACACCGTGAACCCAGGAGGCGGAGGTTGCAGTGAGCTGAGATCACGTTTTAGAGCTAGAAATAGCAA
P2-40bp-chr13-53435625-1R	TTGCTATTTCTAGCTCTAAAACGTGATCTCAGCTCACTGCAACCTCCGCCTCCTGGGTTCACGGTGTTTCGTCCTTTCCACAA
H1-20bp-chr17-14203026-2F	TTGTGGAAAGGACGAAACACCGCCAGGAGTTGGAGGCCCTCGTTTTAGAGCTAGAAATAGCAA
H1-20bp-chr17-14203026-2R	TTGCTATTTCTAGCTCTAAAACGAGGGCCTCCAACTCCTGGCGGTGTTTCGTCCTTTCCACAA
H1-46bp-chr17-14203026-1F	TTGTGGAAAGGACGAAACACCGTGTTCTGTCCCCGGAACCGCTTGCAGCCAGGAGTTGGAGGCCCTCGTTTTAGAGCTAGAAATAGCAA
H1-46bp-chr17-14203026-1R	TTGCTATTTCTAGCTCTAAAACGAGGGCCTCCAACTCCTGGCTGCAAGCGGTTCCGGGGACAGAACACGGTGTTTCGTCCTTTCCACAA
P1-20bp-chr6-3730356-2F	TTGTGGAAAGGACGAAACACCGGGCCGGGCCTGAGACAGCAGTTTTAGAGCTAGAAATAGCAA
P1-20bp-chr6-3730356-2R	TTGCTATTTCTAGCTCTAAAACTGCTGTCTCAGGCCCGGCCCGGTGTTTCGTCCTTTCCACAA
P1-44bp-chr6-3730356-1F	TTGTGGAAAGGACGAAACACCGGAGTCTAAGCGAAGTCCCTCTCTGGGCCGGGCCTGAGACAGCAGTTTTAGAGCTAGAAATAGCAA
P1-44bp-chr6-3730356-1R	TTGCTATTTCTAGCTCTAAAACTGCTGTCTCAGGCCCGGCCCAGAGAGGGACTTCGCTTAGACTCCGGTGTTTCGTCCTTTCCACAA

Successful insertion was validated by Sanger sequencing. The vectors were transiently transfected into HEK293T cells (ATCC 293T/17, CRL-11268) grown in DMEM (Life Technologies) supplemented with 10 % FBS (Life Technologies). 5x10^5 cells were seeded in 6-well plates the day before, and transfected with 2.5 µg vector using 5 µl Lipofectamine LTX (Life Technologies). Cells were harvested 48 hours after transfection, DNA was extracted using the DNeasy blood and tissue kit (QIAGEN) and targeting site amplified using 2X Phusion High-Fidelity PCR Master Mix with GC Buffer (NEB). Reactions were set up in a 50 µl volume with 2 µl of each primer at a dilution of 10 µM and adding 100 ng input DNA. Amplification program: 1 cylce at 98°C for 30s, 12 cycles of 98°C for 10s, optimised annealing temperature from table below for 10s, 72°C for 10s and final elongation at 72°C for 10 min.

Name	Sequence	AT
Target-P3-1F	TCGTCGGCAGCGTCAGATGTGTATAAGAGACAGGCCACGCCTAGCTACATTTTTG	66°C
Target-P3-1R	GTCTCGTGGGCTCGGAGATGTGTATAAGAGACAGGAAGGAAGGAAGGAAGGAGGAA	
Target-Z1-1F	TCGTCGGCAGCGTCAGATGTGTATAAGAGACAGATGAGACTTGGAGGTTCAGATTCC	68°C
Target-Z1-1R	GTCTCGTGGGCTCGGAGATGTGTATAAGAGACAGGTCAGATTGGTGTGTGTGTGAGAG	
Target-P2-1F	TCGTCGGCAGCGTCAGATGTGTATAAGAGACAGTCCCTCATCTCTTCTAACCATCAG	64°C
Target-P2-1R	GTCTCGTGGGCTCGGAGATGTGTATAAGAGACAGCAACATGGTGAAATCCCATATCTAC	
Target-H1-1F	TCGTCGGCAGCGTCAGATGTGTATAAGAGACAGCCGGTGAGTCACTTCGTGAG	65°C
Target-H1-1R	GTCTCGTGGGCTCGGAGATGTGTATAAGAGACAGTCAAATTCTTACTGGTCGTGTTCA	
Target-P1-1F	TCGTCGGCAGCGTCAGATGTGTATAAGAGACAGAATCCTTCCTTAATTGCCTGTGAC	68°C
Target-P1-1R	GTCTCGTGGGCTCGGAGATGTGTATAAGAGACAGGAGCTTCTCAGGGACCATCTTTAG	

Reactions were cleaned up using Agencourt Ampure beads (Beckman Coulter) at a sample:beads ratio of 1:1.5. In order to prepare libraries for sequencing on the Illumina MiSeq platform, Illumina Nextera indexed adapter sequences were added in a second round of amplification using KAPA HiFi PCR kit (Kapa Biosystems) in a 25 µl reaction with Kapa GC Buffer, 0.75 µl dNTP mix, 0.5 µl KAPA HiFi polymerase, 0.75 µl i5-indexed forward primer (10 µM) and

0.75 µl i7-indexed reverse primer (10 µM) and half the cleaned-up product of the first round of amplification. Cycling conditions were: 1 cycle at 95,◦ C for 5 min, 6 cycles of 98◦C for 20 s, 60◦C for 15 s, 72◦C for 30 s and 1 cycle at 72 ◦C for 5 min. Products were purified using Agencourt Ampure beads (Beckman Coulter) at a sample:beads ratio of 1:0.8. Libraries were quantified using the Quant-iT PicoGreen dsDNA assay (Thermo Fisher) and by qPCR using the Kapa Library Quantification kit (Roche). Libraries were sequenced on the Illumina MiSeq platform according to the manufacturer's instruction. The sequencing data was analyzed using an available pipeline for scoring indels resulting from Cas9-mediated mutagenesis (https://github.com/UCL-BLIC/crispr-parsr/releases/tag/v0.2.1). The pipeline was run using standard parameters except for addition of the "allow-any" flag. In short, CRISPR-parsr aligns the sequenced amplicons to the target locus using Bowtie2 [2] and parses the alignments to classify the sequences as WT (wild-type), INS (insertion), DEL (deletion) or COM (complex). Complex cases relate to cases where more than one insertion or deletion has happened. The sequence highlighted with capital letters correspond to the target of the guide RNA. For insertions and complex events, the '>' '<' mark the location of the event.

Functional validation of gRNAs longer than 30 bp that target YFP

gRNAs targeting YFP (sequences below) were cloned into px459 (pSpCas9(BB)-2A-Puro, Addgene plasmid 48139) using Gibson cloning.

Name	Sequence
gRNA Y1_35bp_F	TCTTGTGGAAAGGACGAAACACCGGTGCCCATCCTGGTCGAGCTGGACGCGACGTAAAGTTTTAGAGCTAGAAATAGCAAGTTAAAATAAGGCT
gRNA Y1_35bp_R	AGCCTTATTTTAACTTGCTATTTCTAGCTCTAAAACTTTACGTCGCGTCCAGCTCGACCAGGATGGGCACCGGTGTTTCGTCCTTTCCACAAGA
gRNA Y1_40bp_F	TCTTGTGGAAAGGACGAAACACCGGGGTGGTGCCCATCCTGGTCGAGCTGGACGCGACGTAAAGTTTTAGAGCTAGAAATAGCAAGTTAAAATAAGGCT

gRNA Y1_40bp_R	AGCCTTATTTTAACTTGCTATTTCTAGCTCTAAAACTTTACGTCGCGTCCAGCTCGACCAGGATGGGCACCACCCCGGTGTTTCGTCCTTTCCACAAGA
gRNA Y2_40bp_F	TCTTGTGGAAAGGACGAAACACCGAGCTGGACGCGACGTAAACGGCCACAAGTTCAGCGTGTCGTTTTAGAGCTAGAAATAGCAAGTTAAAATAAGGCT
gRNA Y2_40bp_R	AGCCTTATTTTAACTTGCTATTTCTAGCTCTAAAACGACACGCTGAACTTGTGGCCGTTTACGTCGCGTCCAGCTCGGTGTTTCGTCCTTTCCACAAGA

Successful insertion was validated by Sanger sequencing. The resulting vectors were transiently transfected into mouse neural stem cells containing a constitutively expressed YFP transgene under the control of a CAG promoter, established from a mouse line [4] and cultured as described before [5, 6]. Cells in 6 well plates at 60% confluence were transfected with 2.5 µg plasmid using 9 µl Lipofectamine 2000 (Life Technologies). After 7 days, cells were detached with Accutase and stained for viability before loss of YFP expression was analysed by flow-cytometry.

Supplementary References:

1. Gilks WR, Richardson S, Spiegelhalter DJ: **Markov chain Monte Carlo in practice**. Boca Raton, Fla.: Chapman & Hall; 1998.
2. Langmead B, Trapnell C, Pop M, Salzberg SL: **Ultrafast and memory-efficient alignment of short DNA sequences to the human genome**. *Genome Biol* 2009, **10**(3):R25.
