# Supplementary figures and images for: CORALINA: a universal method for the generation of gRNA libraries for CRISPR-based screening
Source: BMC Genomics. 2016 Nov 14;17:917. doi: 10.1186/s12864-016-3268-z (PMC5109649; doi:10.1186/s12864-016-3268-z)

**A**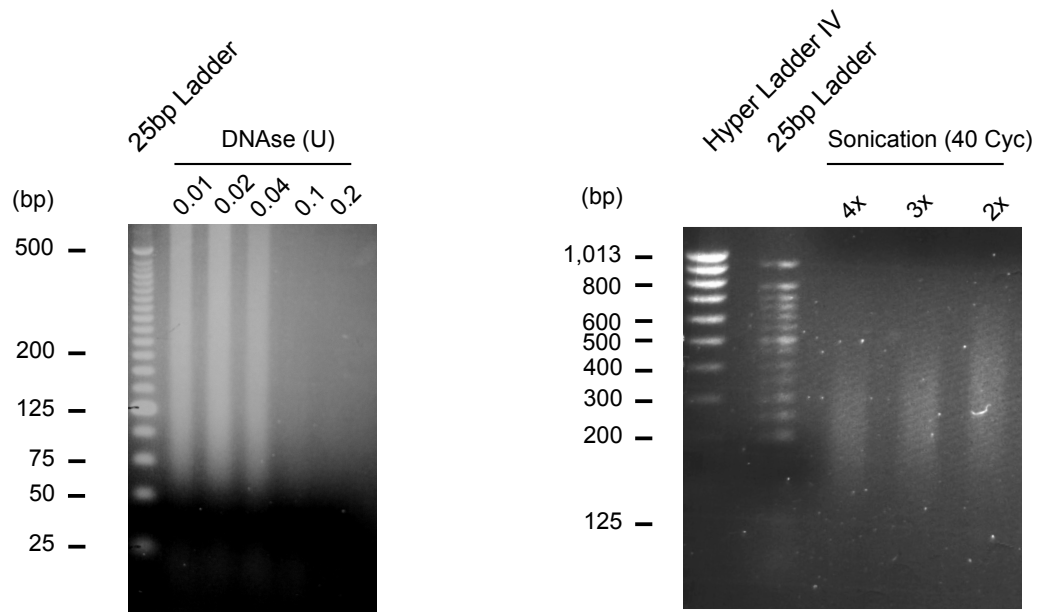**B**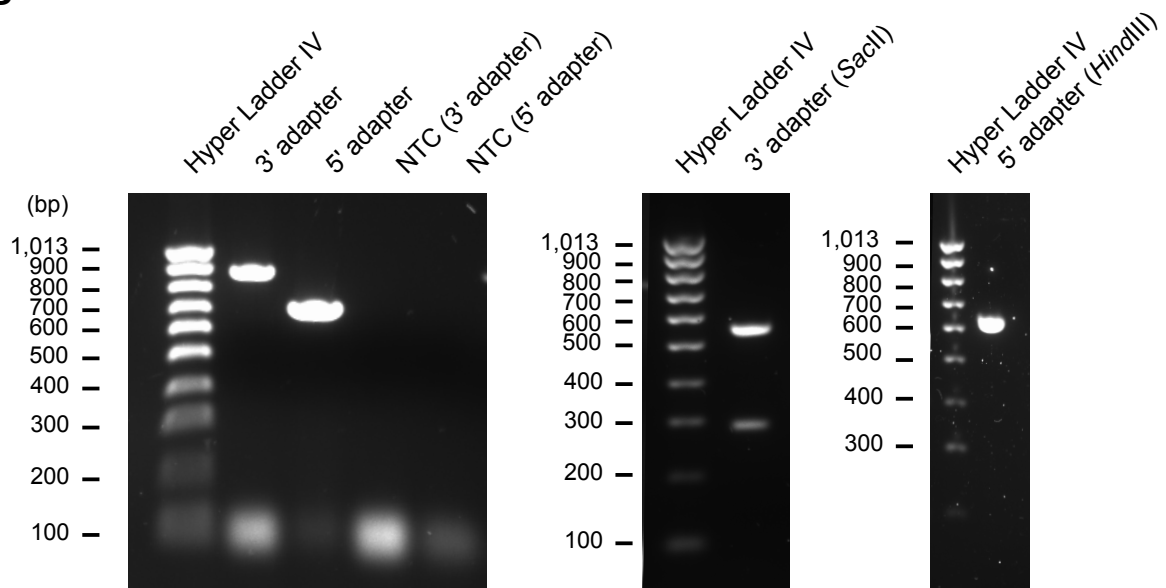

Supplement: Additional file 2: Figure S1. — (A) DNAse digestion (left) or sonication (right) has proven inappropriate for the controlled and efficient generation small (<30 bp) DNA fragments from genomic DNA. (B) Overhang adapters enabling Gibson assembly have been generated using PCR (left side) and cut (right side) to produce asymmetric linker ends (see Fig. 1). (PDF 2811 kb) [file 12864_2016_3268_MOESM2_ESM.pdf]

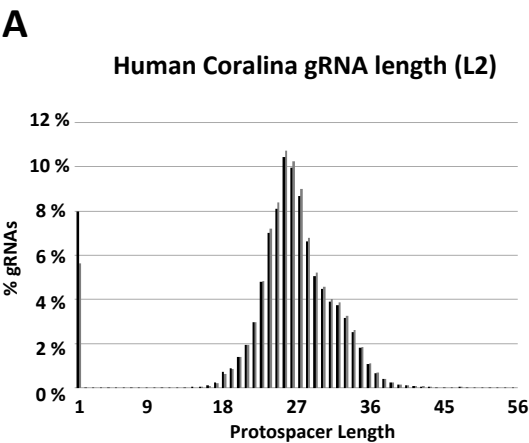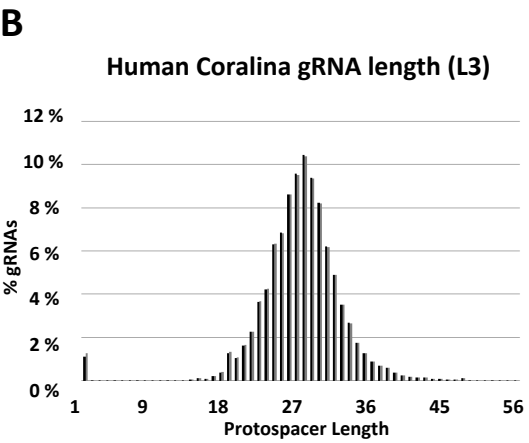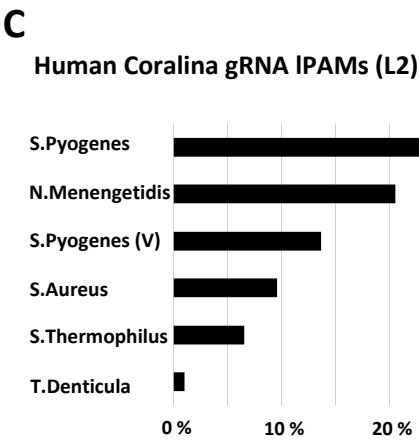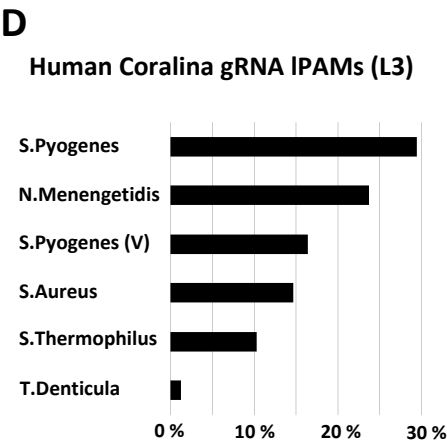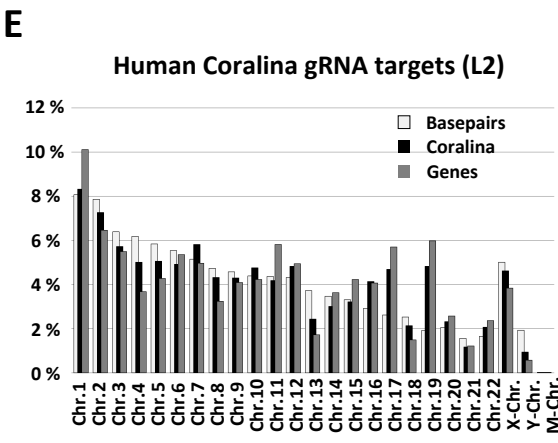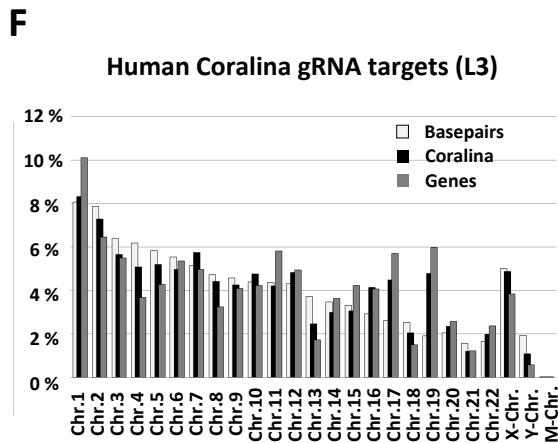

Supplement: Additional file 3: Figure S2. — Analysis of CORALINA gRNAs. Next generation sequencing of CORALINA libraries have been used to analyze generated libraries. (A, B) Quantification of protospacer length of human L2 (A, C, E) and L3 (B, D, F) gRNA libraries. Protospacers are mostly between 18 and 36 bp long. Shown are protospacer lengths detected by forward (black) and reverse (grey) sequencing reads. (C, D) Proportions of CORALINA gRNAs aligning to genomic sites containing functional PAMs of published Cas9 proteins. (E, F) Distribution of sequenced gRNA protospacer alignments to the human genome. While the number of associated CORALINA gRNAs (black bars) is overall correlating to the size of the respective chromosome (white bars), GC- and gene-rich chromosomes (grey bars, 16, 17, 19) are overrepresented relative to their size. (PDF 70 kb) [file 12864_2016_3268_MOESM3_ESM.pdf]

**A**

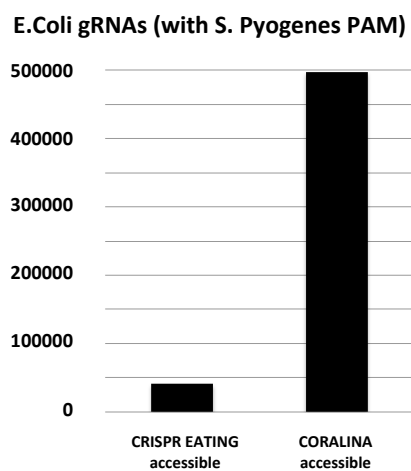

**B**

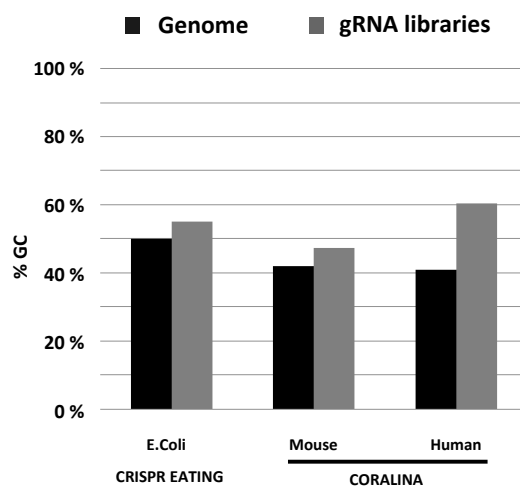

**C**

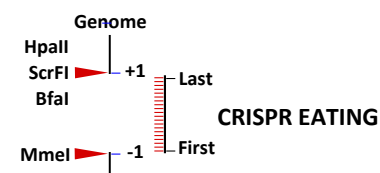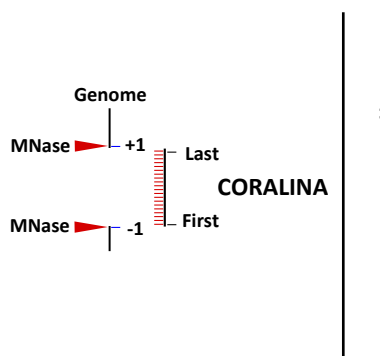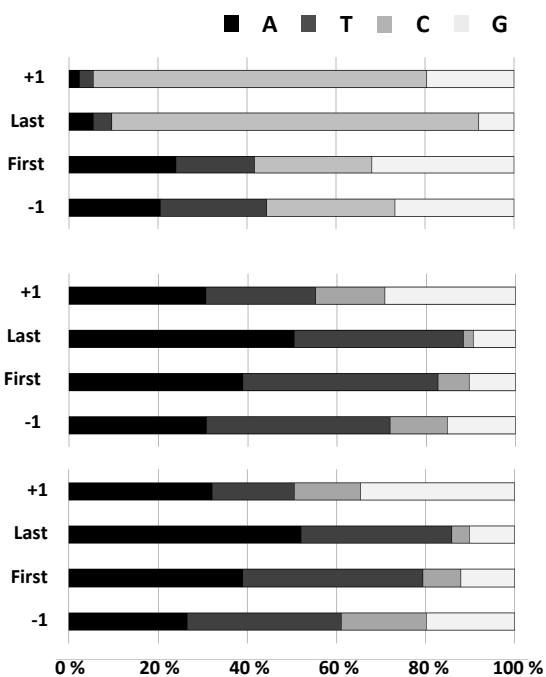

Supplement: Additional file 6: Figure S5. — Comparison of CORALINA libraries to an alternative method used for the generation of large-scale gRNA libraries targeting the E.coli genome (CRISPR-EATING) [29]. (A) Theoretical number of E.coli gRNAs with S.Pyogenes PAM sequences accessible to CRISPR-EATING and CORALINA respectively. (B) GC content of the different gRNA libraries and of the genomes they were generated from. (C) Nucleotide frequency at the 5′ and 3′ ends of the gRNAs incorporated into libraries corresponding to the cutting sites of the nucleases/restriction enzymes used. (PDF 40 kb) [file 12864_2016_3268_MOESM6_ESM.pdf]

**A**

## Human Coralina Library (L2)

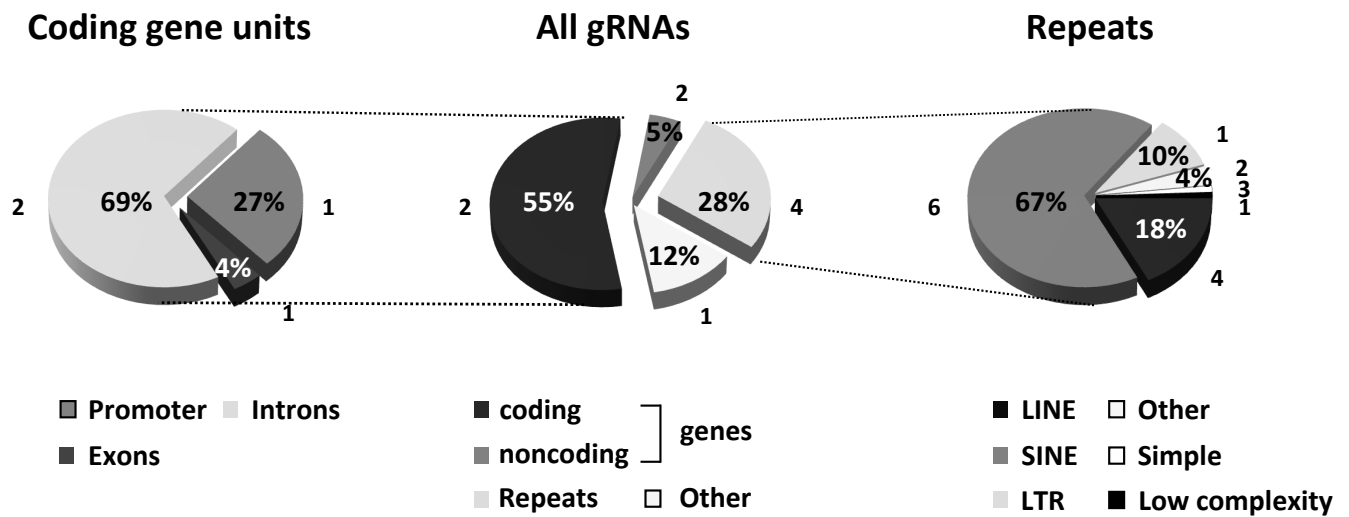

**B**

## Human Coralina Library (L3)

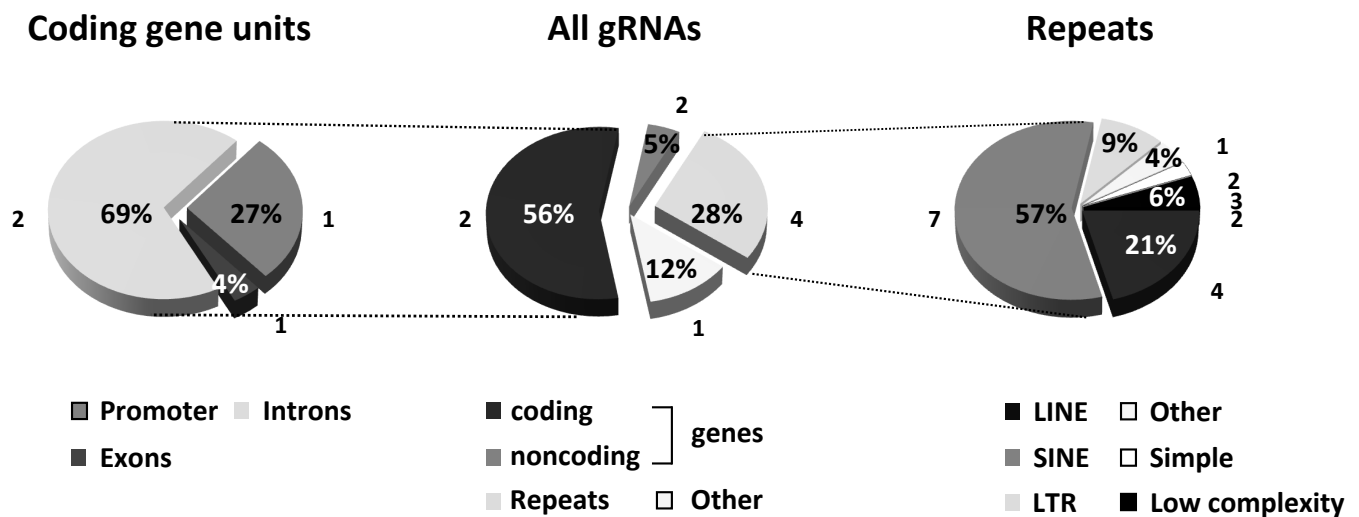

Supplement: Additional file 7: Figure S3. — Analysis of CORALINA libraries. Bowtie has been used to classify targeting sites of gRNAs derived from human (L2, A or L3, B) CORALINA libraries. Pie charts indicating relative proportion of functional domains bound by gRNAs (middle). gRNA protospacer aligning to coding gene units (left) or repeats (right) are further sub-classified. Promoters are defined here as genomic sequences 10 kb upstream the transcriptional start site. Coding and noncoding gene and repeat information has been derived from UCSC. Numbers next to the sectors depict the median number of genomic alignments for the selected group of gRNAs. (PDF 73 kb) [file 12864_2016_3268_MOESM7_ESM.pdf]
