# Supplementary material for: CORALINA: a universal method for the generation of gRNA libraries for CRISPR-based screening
Source: BMC Genomics. 2016 Nov 14;17:917. doi: 10.1186/s12864-016-3268-z (PMC5109649; doi:10.1186/s12864-016-3268-z)
Supplement: Additional file 4: Figure S4. — Functional analysis of CORALINA gRNAs. (A) List of most frequently sequenced alteration generated with gRNA P1-20. (B-E) Top: Schematic depicting CORALINA-derived gRNAs targeting regions in or near various human genes (HS3ST3B1 gRNA H1-46, 46 bp protospacer, PCDH8, gRNA P2- 40, 40 bp protospacer, ZNF790, gRNA Z1-35, 35 bp protospacer, PIK3AP1, gRNA P3-35, 35 bp protospacer). Control gRNAs have been shortened from the 5′ end to yield a 20 bp protospacer. Right: Bargraph depicting percentage of NGS reads displaying indels after targeting wild-type Cas9 using CORALINA-derived gRNAs in HEK293T cells. Below: List of the most frequently sequenced alterations generated by CORALINA and control gRNAs. (PDF 320 kb) [file 12864_2016_3268_MOESM4_ESM.pdf]

A

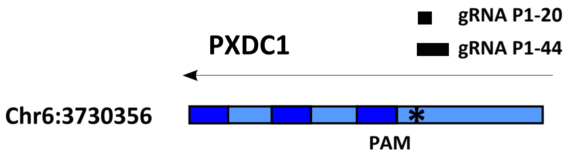


```
                                                                            PAM
                                                                           <--                                                 Type Length
no gRNA
tgtggtttaattatttgcaaggctggctgggccctgaaggacactaaggtatgtctggtgcctcctgcatggacctTGCTGTCTCAGGCCCGGCCCAGAGAGGGACTTCGCTTAGACT WT 0

gRNA P1-20
tgtggtttaattatttgcaaggctggctgggccctgaaggacactaaggtatgtctggtgcctcctgcatggacctTGCTGTCTCAGGCCCGGCCCAGAGAGGGACTTCGCTTAGACT WT 0
tgtggtttaattatttgcaaggctggctgggccctgaaggacactaaggtatgtctggtgcctcctgcatggacctTG----CTCAGGCCCGGCCCAGAGAGGGACTTCGCTTAGACT DEL 4
tgtggtttaattatttgcaaggctggctgggccctgaaggacactaaggtatgtctggtgcctcctgcatggacctTGC--TCTCAGGCCCGGCCCAGAGAGGGACTTCGCTTAGACT DEL 2
tgtggtttaattatttgcaaggctggctgggccctgaaggacactaaggtatgtctggtgcctcctgcatggacctT---------------GCCCAGAGAGGGACTTCGCTTAGACT DEL 15
tgtggtttaattatttgcaaggctggctgggccctgaaggacactaaggtatgtctggtgcctcctgcatggacctTGCT---TCAGGCCCGGCCCAGAGAGGGACTTCGCTTAGACT DEL 3
tgtggtttaattatttgcaaggctggctgggccctgaaggacactaaggtatgtctggtgcctcctgcatggacctTGCT-TCTCAGGCCCGGCCCAGAGAGGGACTTCGCTTAGACT DEL 1
tgtggtttaattatttgcaaggctggctgggccctgaaggacactaaggtatgtctggtgcctcctgcatggacctTGC-GTCTCAGGCCCGGCCCAGAGAGGGACTTCGCTTAGACT DEL 1
tgtggtttaattatttgcaaggctggctgggccctgaaggacactaaggtatgtctggtgcctcctgcatggacct---TGTCTCAGGCCCGGCCCAGAGAGGGACTTCGCTTAGACT DEL 3
tgtggtttaattatttgcaaggctggctgggccctgaaggacactaaggtatgtctggtgcctcctgcat-----------------------CCCAGAGAGGGACTTCGCTTAGACT DEL 23
tgtggtttaattatttgcaaggctggctgggccctgaaggacactaaggtatgtctggtgcctcctgcatggacctTGCT------------GCCCAGAGAGGGACTTCGCTTAGACT DEL 12
tgtggtttaattatttgcaaggctggctgggccctgaaggacactaaggtatgtctggtgcctcctgcatggacctTGCT----------CGGCCCAGAGAGGGACTTCGCTTAGACT DEL 10
tgtggtttaattatttgcaaggctggctgggccctgaaggacactaaggtatgtctggtgcctcctgcat---------TGTCTCAGGCCCGGCCCAGAGAGGGACTTCGCTTAGACT DEL 9
tgtggtttaattatttgcaaggctggctgggccctgaaggacactaaggtatgtctggtgcctcctgcatggacctT--TGTCTCAGGCCCGGCCCAGAGAGGGACTTCGCTTAGACT DEL 2
tgtggtttaattatttgcaaggctggctgggccctgaaggacactaaggtatgtctggtgcctcctgcatgga-------GTCTCAGGCCCGGCCCAGAGAGGGACTTCGCTTAGACT DEL 7
tgtggtttaattatttgcaaggctggctgggccctgaaggacactaaggtatgtctggtgcctcctgcatgg-------TGTCTCAGGCCCGGCCCAGAGAGGGACTTCGCTTAGACT DEL 7
tgtggtttaattatttgcaaggctggctgggccctgaaggacactaaggtatgtctggtgcctcctgcatgga-----CTGTCTCAGGCCCGGCCCAGAGAGGGACTTCGCTTAGACT DEL 5
tgtggtttaattatttgcaaggctggctgggccctgaaggacactaaggtatgtctggtgcctcctgcatggacctTGC---CTCAGGCCCGGCCCAGAGAGGGACTTCGCTTAGACT DEL 3
tgtggtttaattatttgcaaggctggctgggccctgaaggacactaaggtatgtctggtgcctcct--------------------------GCCCAGAGAGGGACTTCGCTTAGACT DEL 26
tgtggtttaattatttgcaaggctggctgggccctgaaggacactaaggtatgtctggtgcct-------------------------CCCGGCCCAGAGAGGGACTTCGCTTAGACT DEL 25
tgtggtttaattatttgcaaggctggctgggccctgaaggacactaaggtatgtctggtgcctcctgcatggacctTG><GTCTCAGGCCCGGCCCAGAGAGGGACTTCGCTTAGACT INS 1
tgtggtttaattatttgcaaggctggctgggccctgaaggacactaaggtatgtctggtgcctcctgcatggacctTG><GTCTCAGGCCCGGCCCAGAGAGGGACTTCGCTTAGACT INS 2
tgtggtttaattatttgcaaggctggctgggccctgaaggacactaaggtatgtctggtgcctcctgcatggacctTG><GTCTCAGGCCCGGCCCAGAGAGGGACTTCGCTTAGACT INS 2
tgtggtttaattatttgcaaggctggctgggccctgaaggacactaaggtatgtctggtgcctcctgcatggacctTG><GTCTCAGGCCCGGCCCAGAGAGGGACTTCGCTTAGACT INS 3
tgtggtttaattatttgcaaggctggctgggccctgaaggacactaaggtatgtctggtgcctcctgcatggacctTG><GTCTCAGGCCCGGCCCAGAGAGGGACTTCGCTTAGACT INS 2
tgtggtttaattatttgcaaggctggctgggccctgaaggacactaaggtatgtctggtgcctcctgcatggacctT><TGTCTCAGGCCCGGCCCAGAGAGGGACTTCGCTTAGACT INS 1
tgtggtttaattatttgcaaggctggctgggccctgaaggacactaaggtatgtctggtgcctcctgcatggacctT><TGTCTCAGGCCCGGCCCAGAGAGGGACTTCGCTTAGACT INS 2
tgtggtttaattatttgc>-------------------------------------------------------------<TCTCAGGCCCGGCCCAGAGAGGGACTTCGCTTAGACT COM 61
tgtggtttaattatttgcaaggctggctgggccc>-------------------------------------------------<AGGCCCGGCCCAGAGAGGGACTTCGCTTAGACT COM 49
tgtggtttaattatttgcaaggctggctgggccc>---------------------------------------------<TCTCAGGCCCGGCCCAGAGAGGGACTTCGCTTAGACT COM 45
tgtggtttaattatttgcaaggctggctgggccctgaaggacacta>---------------------------------<TCTCAGGCCCGGCCCAGAGAGGGACTTCGCTTAGACT COM 33
tgtggtttaattatttgcaaggctggctgggccctgaaggacactaaggtatgtctggtgcctcctgcatggacctTGC>--------------<CAGAGAGGGACTTCGCTTAGACT COM 14
tgtg>---------------------------------------------------------------------------<TCTCAGGCCCGGCCCAGAGAGGGACTTCGCTTAGACT COM 75
tgtggtttaattatttgcaaggctg>-----------------------------------------------------------------------<AGAGGGACTTCGCTTAGACT COM 71
tgtggtttaattatttgcaaggctggctgggccctgaaggacactaaggtatgtctggtgcctcctgcatggacctTGC>-------<CCCGGCCCAGAGAGGGACTTCGCTTAGACT COM 7
tgtggtttaattatttgcaaggctggctgggccctgaaggacactaaggtatgtctggtgcctcctgcatggacctTGC>-------<CCCGGCCCAGAGAGGGACTTCGCTTAGACT COM 7
tgtggtttaattatttgcaagg>--------------------------------------------------------------------<GCCCAGAGAGGGACTTCGCTTAGACT COM 68
tgtggtttaattatt>----------------------------------------------------------------<TCTCAGGCCCGGCCCAGAGAGGGACTTCGCTTAGACT COM 64
tgtggtttaattattt>---------------------------------------------------------------<TCTCAGGCCCGGCCCAGAGAGGGACTTCGCTTAGACT COM 63
tgtggtttaattatttgc>-------------------------------------------------------------<TCTCAGGCCCGGCCCAGAGAGGGACTTCGCTTAGACT COM 61
tgtggtttaattatttgcaaggctggctgggccctgaaggacactaaggtatgtctggtgcctcctgcatggacctTGC>------<GCCCGGCCCAGAGAGGGACTTCGCTTAGACT COM 6
tgtggtttaattatttgcaaggctggctgggccctgaaggacactaaggtatgtctggtgcctcctgcatgga>------<TCTCAGGCCCGGCCCAGAGAGGGACTTCGCTTAGACT COM 6
tgtggtttaattatttgcaa>-----------------------------------------------------------<TCTCAGGCCCGGCCCAGAGAGGGACTTCGCTTAGACT COM 59
tgtggtttaattatttgcaaggctg>------------------------------------------------------<TCTCAGGCCCGGCCCAGAGAGGGACTTCGCTTAGACT COM 54
tgtggtttaattatttgcaaggctg>------------------------------------------------------<TCTCAGGCCCGGCCCAGAGAGGGACTTCGCTTAGACT COM 54
```

**B**

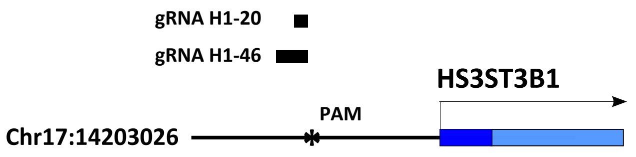


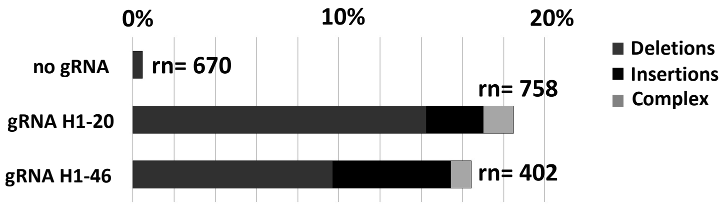


## no gRNA

PAM (arrow) — Type, Length

```
cgtgagtgacactcagccccgggggtcgcgctggggtcggagccggaaggaaTGTTCTGTCCCCGGAACCGCTTGCAGCCAGGAGTTGGAGGCCCTCtggccccgcagaggctgggttgcaggcatcgaaaac WT  0
cgtgagtgacactcagcccc-ggggtcgcgctggggtcggagccggaaggaaTGTTCTGTCCCCGGAACCGCTTGCAGCCAGGAGTTGGAGGCCCTCtggccccgcagaggctgggttgcaggcatcgaaaac DEL 1
cgtgagtgacactcagccccgggggtcgcgctggggtcggagccggaaggaaTGTTCTGTCCCCGGAACCGCTTGCAGCCAGGAGTTGGAGGCCCTCtggccccgcagaggctgggttgcaggcatcg-aaac DEL 1
```

## gRNA H1-46

```
cgtgagtgacactcagccccgggggtcgcgctggggtcggagccggaaggaaTGTTCTGTCCCCGGAACCGCTTGCAGCCAGGAGTTGGAGGCCCTCtggccccgcagaggctgggttgcaggcatcgaaaac WT  0
cgtgagtgacactcagccccgggggtcgcgctggggtcggagccggaaggaaTGTTCTGTCCCCGGAACCGCTTGCAGCCAGGAGTTGGAGG-CCTCtggccccgcagaggctgggttgcaggcatcgaaaac DEL 1
cgtgagtgacactcagccccgggggtcgcgctggggtcggagccggaaggaaTGTTCTGTCCCCGGAACCGCTTGCAGCCAGGAGTTGGA--------ggccccgcagaggctgggttgcaggcatcgaaaac DEL 8
cgtgagtgacactcagccccgggggtcgcgctggggtcggagccggaaggaaTGTTCTGTCCCCGGAACCGCTTGCA---------------------------gcagaggctgggttgcaggcatcgaaaac DEL 27
cgtgagtgacactcagccccgggggtcgcgctggggtcggagccggaaggaaTGTTCTGTCCCCGGAACCGCTTGCAGCCAGGAGTTG------CTCtggccccgcagaggctgggttgcaggcatcgaaaac DEL 6
cgtgagtgacactcagccccgggggtcgcgctggggtcggagccggaaggaaTGTTCTGTCCCCGGAACCGCTTGCAGCCAG-------------------------gaggctgggttgcaggcatcgaaaac DEL 25
cgtgagtgacactcagccccgggggtcgcgctggggtcggagccggaaggaaTGTTCTGTCCCCGGAACCGCTTGCAGCCAGGAGTTGG--------------cgcagaggctgggttgcaggcatcgaaaac DEL 14
cgtgagtgacactcagccccgggggtcgcgctggggtcggagccggaaggaaTGTTCTGTCCCCGGAACCGCTTGCAGCCAGGAGTTGGAGG---------cccgcagaggctgggttgcaggcatcgaaaac DEL 9
cgtgagtgacactcagccccgggggtcgcgctggggtcggagccggaaggaaTGTTCTGTCCCCGGAACCGCTTGCAGCCAGGAGTTGGAGGC-------ccccgcagaggctgggttgcaggcatcgaaaac DEL 7
cgtgagtgacactcagccccgggggtcgcgctggggtcggagccggaaggaaTGTTCTGTCCCCGGAACCGCTTGCAGCCAGGAGTTGG-----CTCtggccccgcagaggctgggttgcaggcatcgaaaac DEL 5
cgtgagtgacactcagccccgggggtcgcgctggggtcggagccggaaggaaTGTTCTGTCCCCGGAACCGCTTGCAGCCAGGAGTTGGAG---CTCtggccccgcagaggctgggttgcaggcatcgaaaac DEL 3
cgtgagtgacactcagccccgggggtcgcgctggggtcggagccggaaggaaTGTTCTGTCCCCGGAACCGCTTGCA-----------------CTCtggccccgcagaggctgggttgcaggcatcgaaaac DEL 17
cgtgagtgacactcagccccgggggtcgcgctggggtcggagccggaaggaaTGTTCTGTCCCCGGAACCGCTTGCA--------------GCCCTCtggccccgcagaggctgggttgcaggcatcgaaaac DEL 14
cgtgagtgacactcagccccgggggtcgcgctggggtcggagccggaaggaaTGTTCTGTCCCCGGAACCGCTTGCAGC-------------CCCTCtggccccgcagaggctgggttgcaggcatcgaaaac DEL 13
cgtgagtgacactcagccccgggggtcgcgctggggtcggagccggaaggaaTGTTCTGTCCCCGGAACCGCTTGCAGCCAGGAGTTGGAGGC------------cagaggctgggttgcaggcatcgaaaac DEL 12
cgtgagtgacactcagccccgggggtcgcgctggggtcggagccggaaggaaTGTTCTGTCCCCGGAACCGCTTGCAGCCAGGA----------CTCtggccccgcagaggctgggttgcaggcatcgaaaac DEL 10
cgtgagtgacactcagccccgggggtcgcgctggggtcggagccggaaggaaTGTTCTGTCCCCGGAACCGCTTGCAGCCAGGAGTTGGAG><CCTCtggccccgcagaggctgggttgcaggcatcgaaaac INS 1
cgtgagtgacactcagccccgggggtcgcgctggggtcggagccggaaggaaTGTTCTGTCCCCGGAACCGCTTGCAGCCAGGAGTTGGAGGC><TCtggccccgcagaggctgggttgcaggcatcgaaaac INS 1
cgtgagtgacactcagccccgggggtcgcgctggggtcggagccggaaggaaTGTTCTGTCCCCGGAACCGCTTGCAGCCAGGAGTTGGAGGC><TCtggccccgcagaggctgggttgcaggcatcgaaaac INS 1
cgtgagtgacactc>----------------------------------------------------------------------------------------<gcagaggctgggttgcaggcatcgaaaac COM 88
cgtgagtgacactcagccccgggggtcg>-------------------------------------------------------------------------------<ggctgggttgcaggcatcgaaaac COM 79
cgtgagtgacactcagccccgggggtcgcgctggggtcggagccggaaggaaTGTTCTGTCCCCGGAACCGCTTGCAGCCAGGAGTTGGAGG>---<tggccccgcagaggctgggttgcaggcatcgaaaac COM 3
cgtgagtgacactcagccccgggggtcgcgctggggtcggagccggaaggaaTGTTCTGTCCCCGGAACCGCTTG>-----------------<CTCtggccccgcagaggctgggttgcaggcatcgaaaac COM 17
```

## gRNA H1-20

```
cgtgagtgacactcagccccgggggtcgcgctggggtcggagccggaaggaaTGTTCTGTCCCCGGAACCGCTTGCAGCCAGGAGTTGGAGGCCCTCtggccccgcagaggctgggttgcaggcatcgaaaac WT  0
cgtgagtgacactcagccccgggggtcgcgctggggtcggagccggaaggaaTGTTCTGTCCCCGGAACCGCTTGCAGCCAGGAGTTGGAGG-CCTCtggccccgcagaggctgggttgcaggcatcgaaaac DEL 1
cgtgagtgacactcagccccgggggtcgcgctggggtcggagccggaaggaaTGTTCTGTCCCCGGAACCGCTTGCAGCCAGGAGTTGGAGG--CTCtggccccgcagaggctgggttgcaggcatcgaaaac DEL 2
cgtgagtgacactcagccccgggggtcgcgctggggtcggagccggaaggaaTGTTCTGTCCCCGGAACCGCTTGCAGCCAGGAGTTGGA--------ggccccgcagaggctgggttgcaggcatcgaaaac DEL 8
cgtgagtgacactcagccccgggggtcgcgctggggtcggagccggaaggaaTGTTCTGTCCCCGGAACCGCTTGCAGCCAGGAGTTGGAGG---------cccgcagaggctgggttgcaggcatcgaaaac DEL 9
cgtgagtgacactcagccccgggggtcgcgctggggtcggagccggaaggaaTGTTCTGTCCCCGGAACCGCTTGCAGCCAGGAGTTGGAGGCC--Ctggccccgcagaggctgggttgcaggcatcgaaaac DEL 2
cgtgagtgacactcagccccgggggtcgcgctggggtcggagccggaaggaaTGTTCTGTCCCCGGAACCGCTTGCAGCCAGGAGTTG------CTCtggccccgcagaggctgggttgcaggcatcgaaaac DEL 6
cgtgagtgacactcagccccgggggtcgcgctggggtcggagccggaaggaaTGTTCTGTCCCCGGAACCGCTTGCAGCCAGGAGTTGGAGG----------ccgcagaggctgggttgcaggcatcgaaaac DEL 10
cgtgagtgacactcagccccgggggtcgcgctggggtcggagccggaaggaaTGTTCTGTCCCCGGAACCGCTTGCAGCCAGGAGTTGG-----CTCtggccccgcagaggctgggttgcaggcatcgaaaac DEL 5
cgtgagtgacactcagccccgggggtcgcgctggggtcggagccggaaggaaTGTTCTGTCCCCGGAACCGCTTGCAGCCAGGAGTTGGAGGCC----------------gctgggttgcaggcatcgaaaac DEL 16
cgtgagtgacactcagccccgggggtcgcgctggggtcggagccggaaggaaTGTTCTGTCCCCGGAACCGCTTGCAGCCAGGAG---------CTCtggccccgcagaggctgggttgcaggcatcgaaaac DEL 9
cgtgagtgacactcagccccgggggtcgcgctggggtcggagccggaaggaaTGTTCTGTCCCCGGAACCGCTTGCAGCCAGGAGTTGGAGGC-------ccccgcagaggctgggttgcaggcatcgaaaac DEL 7
cgtgagtgacactcagccccgggggtcgcgctggggtcggagccggaaggaaTGTTCTGTCCCCGGAACCGCTTGCAGCCAGGAGTTGGAGGCC----ggccccgcagaggctgggttgcaggcatcgaaaac DEL 4
cgtgagtgacactcagccccgggggtcgcgctggggtcggagccggaaggaaTGTTCTGTCCCCGGAACCGCTTGCAGCCAGGAGTTGGAGGC---Ctggccccgcagaggctgggttgcaggcatcgaaaac DEL 3
cgtgagtgacactcagccccgggggtcgcgctggggtcggagccggaaggaaTGTTCTGTCCCCGGAACCG-----------------------CTCtggccccgcagaggctgggttgcaggcatcgaaaac DEL 23
cgtgagtgacactcagccccgggggtcgcgctggggtcggagccggaaggaaTGTTCTGTCCCCGGAACCGCTT-------------------CCTCtggccccgcagaggctgggttgcaggcatcgaaaac DEL 19
cgtgagtgacactcagccccgggggtcgcgctggggtcggagccggaaggaaTGTTCTGTCCCCGGAACCGCTTGCAGCCAGGAGTTG-------------------gaggctgggttgcaggcatcgaaaac DEL 19
cgtgagtgacactcagccccgggggtcgcgctggggtcggagccggaaggaaTGTTCTGTCCCCGGAACCGCTTGCAG----------------CTCtggccccgcagaggctgggttgcaggcatcgaaaac DEL 16
cgtgagtgacactcagccccgggggtcgcgctggggtcggagccggaaggaaTGTTCTGTCCCCGGAACCGCTTGCAG---------------CCTCtggccccgcagaggctgggttgcaggcatcgaaaac DEL 15
cgtgagtgacactcagccccgggggtcgcgctggggtcggagccggaaggaaTGTTCTGTCCCCGGAACCGCTTGCAGCCAGGAGTTGGAG><CCTCtggccccgcagaggctgggttgcaggcatcgaaaac INS 1
cgtgagtgacactcagccccgggggtcgcgctggggtcggagccggaaggaaTGTTCTGTCCCCGGAACCGCTTGCAGCCAGGAGTTGGAGGC><TCtggccccgcagaggctgggttgcaggcatcgaaaac INS 2
cgtgagtgacactcagccccgggggtcgcgctggggtcggagccggaaggaaTGTTCTGTCCCCGGAACCGCTTGCAGCCAGGAGTTGGAGGC><TCtggccccgcagaggctgggttgcaggcatcgaaaac INS 1
cgtgagtgacactcagccccgggggtcgcgctggggtcggagccggaaggaaTGTTCTGTCCCCGGAACCGCTTGCAGCCAGGAGTTGGAGG><CTCtggccccgcagaggctgggttgcaggcatcgaaaac INS 2
cgtgagtgacactcagccccgggggtcgcgctggggtcggagccggaaggaaTGTTCTGTCCCCGGAACCGCTTGCAGCCAGGAGTTGGAG><CCTCtggccccgcagaggctgggttgcaggcatcgaaaac INS 2
cgtgagtgacactcagccccgggggtcgcgctggggtcggagccggaaggaaTGTTCTGTCCCCGGAACCGCTTGCAGCCAGGAGTTGGAGGC><TCtggccccgcagaggctgggttgcaggcatcgaaaac INS 1
cgtgagtgacactcagccccgggggtcgcgctggggtcggagccggaaggaaTGTTCTGTCCCCGGAACCGCTTGCAGCCAGGAGTTGGAGGC><TCtggccccgcagaggctgggttgcaggcatcgaaaac INS 1
cgtgagtgacactcagccccgggggtcgcgctggggtcggagccggaaggaaTGTTCTGTCCCCGGAACCGCTTGCAGCCAGGAGTTGGAGGC>---------<gcagaggctgggttgcaggcatcgaaaac COM 9
cgtgagtgacactcagccccgggggtcgcgctggggtcggagccggaaggaaTGTTCTGTCCCCGGAACCGCTTGCAGCCAGGAGTTGGAGG>-------------<gaggctgggttgcaggcatcgaaaac COM 13
cgtgagtgacactcagccccgggggtcgcgctggggtcggagccggaaggaaTGTTCTGTCCCCGGAACCGCTTGCAGCCAGGAGTTGGAGGC>---------<gcagaggctgggttgcaggcatcgaaaac COM 9
cgtgagtgacactcagccccgggggtcgcgctggggtcggagccggaaggaaTG>---------------------------------------------------<gaggctgggttgcaggcatcgaaaac COM 51
cgtgagtgacactcagccccgggggtcgcgctggggtcggagccggaaggaaTGTTCTGTCCC>-------------------------------<Ctggccccgcagaggctgggttgcaggcatcgaaaac COM 31
cgtgagtgacactcagccccgggggtcgcgctggggtcggagccggaaggaaTGTTCTGTCCCCGGAACCGCT>---------------------<Ctggccccgcagaggctgggttgcaggcatcgaaaac COM 21
cgtgagtgacactcagccccgggggtcgcgctggggtcggagccggaaggaaTGTTCTGTCCCCGGAACCGCTTGCAGCCAGGAGTTGGAGG>-------------<gaggctgggttgcaggcatcgaaaac COM 13
cgtgagtgacactcagccccgggggtcgcgctggggtcggagccggaaggaaTGTTCTGTCCCCGGAACCGCTTGCAGCCAGGAGT>-----------<gccccgcagaggctgggttgcaggcatcgaaaac COM 11
cgtgagtgacactcagccccgggggtcgcgctggggtcggagccggaaggaaTGTTCTGTCCCCGGAACCGCTTGCAGCCAGGAGTTGGAG>-<CTCtggccccgcagaggctgggttgcaggcatcgaaaac COM 1
```

**C**

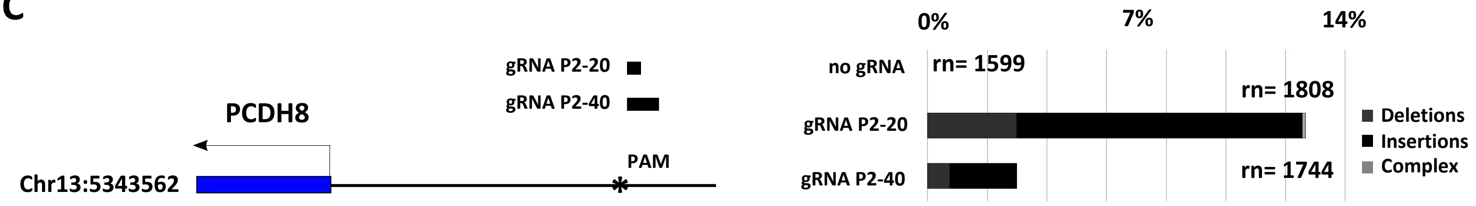


## no gRNA

PAM

```
                                                                                     Type Length
tctatcacccaggctggagtccaGTGATCTCAGCTCACTGCAACCTCCGCCTCCTGGGTTCAagcgattctcctgcctc WT  0
```

## gRNA P2-40

```
tctatcacccaggctggagtccaGTGATCTCAGCTCACTGCAACCTCCGCCTCCTGGGTTCAagcgattctcctgcctc WT  0
tctatcacccaggctggagtccaGTG---------CACTGCAACCTCCGCCTCCTGGGTTCAagcgattctcctgcctc DEL 9
tctatcacccaggctggagtccaGTGA--TCAGCTCACTGCAACCTCCGCCTCCTGGGTTCAagcgattctcctgcctc DEL 2
tctatcacccaggctggagtccaGTG--CTCAGCTCACTGCAACCTCCGCCTCCTGGGTTCAagcgattctcctgcctc DEL 2
tctatcacccaggctggagtcca--GATCTCAGCTCACTGCAACCTCCGCCTCCTGGGTTCAagcgattctcctgcctc DEL 2
tctatcacccaggctggagtccaGT-ATCTCAGCTCACTGCAACCTCCGCCTCCTGGGTTCAagcgattctcctgcctc DEL 1
tctatcacccaggctggagtccaGT-------GCTCACTGCAACCTCCGCCTCCTGGGTTCAagcgattctcctgcctc DEL 7
tctatcacccaggctggagtccaGTG-TCTCAGCTCACTGCAACCTCCGCCTCCTGGGTTCAagcgattctcctgcctc DEL 1
tctatcacccaggctggagtcca----------------GCAACCTCCGCCTCCTGGGTTCAagcgattctcctgcctc DEL 16
tctatcacccaggctggagtccaGT><TCTCAGCTCACTGCAACCTCCGCCTCCTGGGTTCAagcgattctcctgcctc INS 1
```

## gRNA P2-20

```
tctatcacccaggctggagtccaGTGATCTCAGCTCACTGCAACCTCCGCCTCCTGGGTTCAagcgattctcctgcctc WT 0
tctatcacccaggctggagtccaGT-ATCTCAGCTCACTGCAACCTCCGCCTCCTGGGTTCAagcgattctcctgcctc DEL 1
tctatcacccaggctggagtc---------CAGCTCACTGCAACCTCCGCCTCCTGGGTTCAagcgattctcctgcctc DEL 9
tctatcacccaggctggagtccaGTG---------CACTGCAACCTCCGCCTCCTGGGTTCAagcgattctcctgcctc DEL 9
tctatcacccaggctggagtcc----ATCTCAGCTCACTGCAACCTCCGCCTCCTGGGTTCAagcgattctcctgcctc DEL 4
tctatcacccaggctggagtccaGTGA--TCAGCTCACTGCAACCTCCGCCTCCTGGGTTCAagcgattctcctgcctc DEL 2
tctatcacccaggctggagtcca--GATCTCAGCTCACTGCAACCTCCGCCTCCTGGGTTCAagcgattctcctgcctc DEL 2
tctatcacccaggctggagtccaG--------------TGCAACCTCCGCCTCCTGGGTTCAagcgattctcctgcctc DEL 14
tctatcacccaggctggagtccaGTG-------------GCAACCTCCGCCTCCTGGGTTCAagcgattctcctgcctc DEL 13
tctatcacccaggctggagtccaGTGA------------GCAACCTCCGCCTCCTGGGTTCAagcgattctcctgcctc DEL 12
tctatcacccaggctggagtccaGTG----------ACTGCAACCTCCGCCTCCTGGGTTCAagcgattctcctgcctc DEL 10
tctatcacccaggctggagtccaGTGA--------CACTGCAACCTCCGCCTCCTGGGTTCAagcgattctcctgcctc DEL 8
tctatcacccaggctggagtccaGTGA-------TCACTGCAACCTCCGCCTCCTGGGTTCAagcgattctcctgcctc DEL 7
tctatcacccaggctggagtccaGT-------GCTCACTGCAACCTCCGCCTCCTGGGTTCAagcgattctcctgcctc DEL 7
tctatcacccaggctggagt------ATCTCAGCTCACTGCAACCTCCGCCTCCTGGGTTCAagcgattctcctgcctc DEL 6
tctatcacccaggctggagtccaGTG----CAGCTCACTGCAACCTCCGCCTCCTGGGTTCAagcgattctcctgcctc DEL 4
tctatcacccaggctggagtccaG---TCTCAGCTCACTGCAACCTCCGCCTCCTGGGTTCAagcgattctcctgcctc DEL 3
tctatcacccaggctggagtccaGTG--CTCAGCTCACTGCAACCTCCGCCTCCTGGGTTCAagcgattctcctgcctc DEL 2
tctatcacccaggctggagtccaGTGAT-TCAGCTCACTGCAACCTCCGCCTCCTGGGTTCAagcgattctcctgcctc DEL 1
tctatcacccaggctggagtccaGT><TCTCAGCTCACTGCAACCTCCGCCTCCTGGGTTCAagcgattctcctgcctc INS 1
tctatcacccaggctggagtccaGT><TCTCAGCTCACTGCAACCTCCGCCTCCTGGGTTCAagcgattctcctgcctc INS 2
tctatcacccaggctggagtccaGT><TCTCAGCTCACTGCAACCTCCGCCTCCTGGGTTCAagcgattctcctgcctc INS 1
tctatcacccaggctggagtccaGT><TCTCAGCTCACTGCAACCTCCGCCTCCTGGGTTCAagcgattctcctgcctc INS 3
tctatcacccaggctggagtccaGT><TCTCAGCTCACTGCAACCTCCGCCTCCTGGGTTCAagcgattctcctgcctc INS 2
tctatcacccaggctggagtccaGTG>-----<CTCACTGCAACCTCCGCCTCCTGGGTTCAagcgattctcctgcctc COM 5
tctatcacccaggctggagtccaGTG>-------------------<CGCCTCCTGGGTTCAagcgattctcctgcctc COM 19
```

**D**

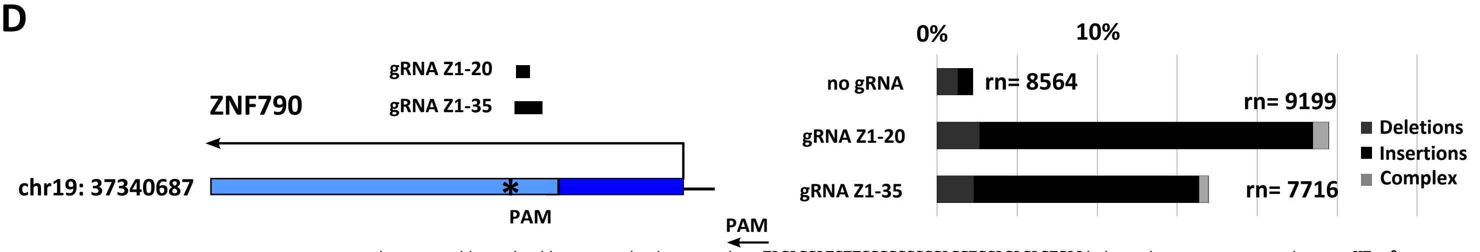


PAM

**no gRNA**

```
cagtgacccacattcaggtgattacacacactcatacacagatccaTACACGATCTTGCCCCCCCCACCTCCACACACTCAAatgacctccccacgcccaaagctgcaca WT  0
tcaggacccacattcaggtgattacacacactcatacacagatccaTACACGATCTTG-CCCCCCCACCTCCACACACTCAAatgacctccccacgcccaaagctgcaca DEL 1
cagtgacccacattcaggtgattacacacactcatacacagatccaTACACGATCTTGCCCCCCCCACCTCCACACACTCAAatgacct-cccacgcccaaagctgcaca DEL 1
cagtgacccacattcaggtgatt--acacactcatacacagatccaTACACGATCTTGCCCCCCCCACCTCCACACACTCAAatgacctccccacgcccaaagctgcaca DEL 2
cagtgacccacattcaggtgattacacacactcatacacagatccaTACACGATCTTG--CCCCCCACCTCCACACACTCAAatgacctccccacgcccaaagctgcaca DEL 2
cagtgacccacattcaggtgattacacacactcatacacagatccaTACACGATCTT><CCCCCCCACCTCCACACACTCAAatgacctccccacgcccaaagctgcaca INS 1
cagtgacccacattcaggtgattacacacactcatacacagatccaTACACGATCTT><CCCCCCCACCTCCACACACTCAAatgacctccccacgcccaaagctgcaca INS 1
cagtgacccacattcaggtgattacacacactcatacacagatccaTACACGATCT><CCCCCCCCACCTCCACACACTCAAatgacctccccacgcccaaagctgcaca INS 1
cagtgacccacattcaggtgattacacacactcatacacagatccaTACACGATCTT>--<CCCCCACCTCCACACACTCAAatgacctccccacgcccaaagctgcaca COM 2
cagtgacccacattcaggtgattacacacactcatacacagatccaTACACGATCTTG>--------------------<AAatgacctccccacgcccaaagctgcaca COM 20
cagtgacccacattcaggtgattacacacactcatacacaga>-----------------<CCCCCACCTCCACACACTCAAatgacctccccacgcccaaagctgcaca COM 17
```

**gRNA Z1-35**

```
cagtgacccacattcaggtgattacacacactcatacacagatccaTACACGATCTTGCCCCCCCCACCTCCACACACTCAAatgacctccccacgcccaaagctgcaca WT  0
cagtgacccacattcaggtgattacacacactcatacacagatccaTACACGATCTTG-CCCCCCCACCTCCACACACTCAAatgacctccccacgcccaaagctgcaca DEL 1
cagtgacccacattcaggtgattacacacactcatacacagatccaT--ACGATCTTGCCCCCCCCACCTCCACACACTCAAatgacctccccacgcccaaagctgcaca DEL 2
cagtgacccacattcaggtgattacacacactcatacacagatccaTA-----------------CACCTCCACACACTCAAatgacctccccacgcccaaagctgcaca DEL 17
cagtgacccacattcaggtgattacacacactcatacacagatccaTACA-GATCTTGCCCCCCCCACCTCCACACACTCAAatgacctccccacgcccaaagctgcaca DEL 1
cagtgacccacattcaggtgattacacacactcatacacagatccaTACA--ATCTTGCCCCCCCCACCTCCACACACTCAAatgacctccccacgcccaaagctgcaca DEL 2
cagtgacccacattcaggtgattacacacactcatacacagatccaTA-ACGATCTTGCCCCCCCCACCTCCACACACTCAAatgacctccccacgcccaaagctgcaca DEL 1
cagtgacccacattcaggtgattacacacactcatacacagat------ACGATCTTGCCCCCCCCACCTCCACACACTCAAatgacctccccacgcccaaagctgcaca DEL 6
cagtgacccacattcaggtgattacacacactcatacacagatccaTA---GATCTTGCCCCCCCCACCTCCACACACTCAAatgacctccccacgcccaaagctgcaca DEL 3
cagtgacccacattcaggtgattacacacactcatacaca-----------GATCTTGCCCCCCCCACCTCCACACACTCAAatgacctccccacgcccaaagctgcaca DEL 11
cagtgacccacattcaggtgattacacacactcatacacagatccaT----GATCTTGCCCCCCCCACCTCCACACACTCAAatgacctccccacgcccaaagctgcaca DEL 4
cagtgacccacattcaggtgattacacacact------------caTACACGATCTTGCCCCCCCCACCTCCACACACTCAAatgacctccccacgcccaaagctgcaca DEL 12
cagtgacccacattcaggtgattacacacactcatacacagat-----CACGATCTTGCCCCCCCCACCTCCACACACTCAAatgacctccccacgcccaaagctgcaca DEL 5
cagtgacccacattcaggtgattacacacactcatacacagatccaTACACGATCTTGCCCCCCCCACCTCCACACACTCAAatgacct-cccacgcccaaagctgcaca DEL 1
cagtgacccacattcaggtgattacacacactcatacacagatccaTACA-----------CCCCCACCTCCACACACTCAAatgacctccccacgcccaaagctgcaca DEL 11
cagtgacccacattcaggtgattacacacactcatacacagatccaTACA--------CCCCCCCCACCTCCACACACTCAAatgacctccccacgcccaaagctgcaca DEL 8
cagtgacccacattcaggtgattacacacactcatacacagatccaTACAC------GCCCCCCCCACCTCCACACACTCAAatgacctccccacgcccaaagctgcaca DEL 6
cagtgacccacattcaggtgattacacacactcatacacagatccaTACA------TGCCCCCCCCACCTCCACACACTCAAatgacctccccacgcccaaagctgcaca DEL 6
cagtgacccacattcaggtgattacacacactcatacacagatccaTACA-----TTGCCCCCCCCACCTCCACACACTCAAatgacctccccacgcccaaagctgcaca DEL 5
cagtgacccacattcaggtgattacacacactcatacacagatccaTA><CGATCTTGCCCCCCCCACCTCCACACACTCAAatgacctccccacgcccaaagctgcaca INS 1
cagtgacccacattcaggtgattacacacactcatacacagatccaTACACGATCTT><CCCCCCCACCTCCACACACTCAAatgacctccccacgcccaaagctgcaca INS 1
cagtgacccacattcaggtgattacacacactcatacacagatccaTA><CGATCTTGCCCCCCCCACCTCCACACACTCAAatgacctccccacgcccaaagctgcaca INS 2
cagtgacccacattcaggtgattacacacactcatacacagatccaTA><CGATCTTGCCCCCCCCACCTCCACACACTCAAatgacctccccacgcccaaagctgcaca INS 2
cagtgacccacattcaggtgattacacacactcatacacagatcca><CACGATCTTGCCCCCCCCACCTCCACACACTCAAatgacctccccacgcccaaagctgcaca INS 2
cagtgacccacattcaggtgattacacacactcatacacagatccaTACACGATCTTGCCCCCC><ACCTCCACACACTCAAatgacctccccacgcccaaagctgcaca INS 1
cagtgacccacattcaggtgattacacacactcatacacagatccaTAC>----------<CCCCCACCTCCACACACTCAAatgacctccccacgcccaaagctgcaca COM 10
cagtgacccacattcaggtgattacacacactcatacacagatccaTAC>---------<CCCCCCACCTCCACACACTCAAatgacctccccacgcccaaagctgcaca COM 9
cagtgacccacattcaggtgattacacacactcatacacagatccaTACA>--------------<ACCTCCACACACTCAAatgacctccccacgcccaaagctgcaca COM 14
cagtgacccacattcaggtgattacacacactcatacacagatccaTACA>---------<CCCCCACCTCCACACACTCAAatgacctccccacgcccaaagctgcaca COM 9
cagtgacccacattcaggtgattacacacactcatacacag>--------<GATCTTGCCCCCCCCACCTCCACACACTCAAatgacctccccacgcccaaagctgcaca COM 8
cagtgacccacattcaggtgattacacacactcatacacagatccaTACACGATCTTG>--------<CTCCACACACTCAAatgacctccccacgcccaaagctgcaca COM 8
cagtgacccacattcaggtgattacacacactcatacacagatccaTAC>------<GCCCCCCCCACCTCCACACACTCAAatgacctccccacgcccaaagctgcaca COM 6
c>---------------------------------------------------<CTTGCCCCCCCCACCTCCACACACTCAAatgacctccccacgcccaaagctgcaca COM 51
cagtgacccacattcaggtgattacacacactcatacacagatcc>----<GATCTTGCCCCCCCCACCTCCACACACTCAAatgacctccccacgcccaaagctgcaca COM 4
cagtgacccacat>----------------------------------------------<CCCCCACCTCCACACACTCAAatgacctccccacgcccaaagctgcaca COM 46
cagtgacccacattcaggtgattacacacactcatacacagatccaTAC>-----------------------------------------<cacgcccaaagctgcaca COM 42
cagtgacccacattcaggtgattacacacactcatacacagatccaTACACGAT>----<CCCCCCACCTCCACACACTCAAatgacctccccacgcccaaagctgcaca COM 4
cagtgacccacattcaggtgattacacacactcatacacagatccaTAC>----<TTGCCCCCCCCACCTCCACACACTCAAatgacctccccacgcccaaagctgcaca COM 4
cagtgaccc>----------------------------------------<GATCTTGCCCCCCCCACCTCCACACACTCAAatgacctccccacgcccaaagctgcaca COM 40
cagtgacccacattcaggtgattacacacactcatacacagatccaT>---------------------------------------<tccccacgcccaaagctgcaca COM 40
cagtgaccca>---------------------------------------<GATCTTGCCCCCCCCACCTCCACACACTCAAatgacctccccacgcccaaagctgcaca COM 37
cagtgacccacat>------------------------------------<GATCTTGCCCCCCCCACCTCCACACACTCAAatgacctccccacgcccaaagctgcaca COM 36
cagtgacccacattcaggtgattacacacactcatacacagatccaTACA>-----------------------------------<ctccccacgcccaaagctgcaca COM 36
```

**gRNA Z1-20**

```
cagtgacccacattcaggtgattacacacactcatacacagatccaTACACGATCTTGCCCCCCCCACCTCCACACACTCAAatgacctccccacgcccaaagctgcaca WT  0
cagtgacccacattcaggtgattacacacactcatacacagatccaTACACGATCTTG-CCCCCCCACCTCCACACACTCAAatgacctccccacgcccaaagctgcaca DEL 1
cagtgacccacattcaggtgattacacacactcatacacagatccaT--ACGATCTTGCCCCCCCCACCTCCACACACTCAAatgacctccccacgcccaaagctgcaca DEL 2
cagtgacccacattcaggtgattacacacactcatacacagatccaTACA--ATCTTGCCCCCCCCACCTCCACACACTCAAatgacctccccacgcccaaagctgcaca DEL 2
cagtgacccacattcaggtgattacacacactcatacaca-----------GATCTTGCCCCCCCCACCTCCACACACTCAAatgacctccccacgcccaaagctgcaca DEL 11
cagtgacccacattcaggtgattacacacactcatacacagatccaTACA----CTTGCCCCCCCCACCTCCACACACTCAAatgacctccccacgcccaaagctgcaca DEL 4
cagtgacccacattcaggtgattacacacactcatacacagatccaTACA-GATCTTGCCCCCCCCACCTCCACACACTCAAatgacctccccacgcccaaagctgcaca DEL 1
cagtgacccacattcaggtgattacacacactcatacacagatc------CGATCTTGCCCCCCCCACCTCCACACACTCAAatgacctccccacgcccaaagctgcaca DEL 6
cagtgacccacattcaggtgattacacacactcatacacagatccaTACA--------CCCCCCCCACCTCCACACACTCAAatgacctccccacgcccaaagctgcaca DEL 8
cagtgacccacattcaggtgattacacacactcatacacagatccaTAC---ATCTTGCCCCCCCCACCTCCACACACTCAAatgacctccccacgcccaaagctgcaca DEL 3
cagtgacccacattcaggtgattacacacactcatacacagatccaTA-ACGATCTTGCCCCCCCCACCTCCACACACTCAAatgacctccccacgcccaaagctgcaca DEL 1
cagtgacccacattcaggtgattacacacactcatacacagatccaTACA---------CCCCCCCACCTCCACACACTCAAatgacctccccacgcccaaagctgcaca DEL 9
cagtgacccacattcaggtgattacacacactcatacacaga-------ACGATCTTGCCCCCCCCACCTCCACACACTCAAatgacctccccacgcccaaagctgcaca DEL 7
cagtgacccacattcaggtgattacacacactcatacacagatccaTACA-------GCCCCCCCCACCTCCACACACTCAAatgacctccccacgcccaaagctgcaca DEL 7
cagtgacccacattcaggtgattacacacactcatacaca----------------------------CTCCACACACTCAAatgacctccccacgcccaaagctgcaca DEL 28
cagtgacccacattcaggtgattacacacactcatacacagatccaTACA---------------CACCTCCACACACTCAAatgacctccccacgcccaaagctgcaca DEL 15
cagtgacccacattcaggtgattacacacactcata-------------ACGATCTTGCCCCCCCCACCTCCACACACTCAAatgacctccccacgcccaaagctgcaca DEL 13
cagtgacccacattcaggtgattacacacactcatacacagatcca------------CCCCCCCCACCTCCACACACTCAAatgacctccccacgcccaaagctgcaca DEL 12
cagtgacccacattcaggtgattacacacactcatacacagatccaTACA------TGCCCCCCCCACCTCCACACACTCAAatgacctccccacgcccaaagctgcaca DEL 6
cagtgacccacattcaggtgattacacacactcatacacagatccaTA><CGATCTTGCCCCCCCCACCTCCACACACTCAAatgacctccccacgcccaaagctgcaca INS 1
cagtgacccacattcaggtgattacacacactcatacacagatccaTACACGATCTT><CCCCCCCACCTCCACACACTCAAatgacctccccacgcccaaagctgcaca INS 1
cagtgacccacattcaggtgattacacacactcatacacagatccaTA><CGATCTTGCCCCCCCCACCTCCACACACTCAAatgacctccccacgcccaaagctgcaca INS 2
cagtgacccacattcaggtgattacacacactcatacacagatccaTA><CGATCTTGCCCCCCCCACCTCCACACACTCAAatgacctccccacgcccaaagctgcaca INS 3
cagtgacccacattcaggtgattacacacactcatacacagatcca><CACGATCTTGCCCCCCCCACCTCCACACACTCAAatgacctccccacgcccaaagctgcaca INS 2
cagtgacccacattcaggtgattacacacactcatacacagatccaTA><CGATCTTGCCCCCCCCACCTCCACACACTCAAatgacctccccacgcccaaagctgcaca INS 2
cagtgacccacattcaggtgattacacacactcatacacagatccaTA><CGATCTTGCCCCCCCCACCTCCACACACTCAAatgacctccccacgcccaaagctgcaca INS 4
cagtgacccacattcaggtgattacacacactcatacacagatccaTA><CGATCTTGCCCCCCCCACCTCCACACACTCAAatgacctccccacgcccaaagctgcaca INS 4
cagtgacccacattcaggtgattacacacactcatacacagatccaTAC><GATCTTGCCCCCCCCACCTCCACACACTCAAatgacctccccacgcccaaagctgcaca INS 1
cagtgacccacattcaggtgattacacacactcatacacagatccaTA><CGATCTTGCCCCCCCCACCTCCACACACTCAAatgacctccccacgcccaaagctgcaca INS 1
cagtgacccacattcaggtgattacacacactcatacacagatccaTAC>----------<CCCCCACCTCCACACACTCAAatgacctccccacgcccaaagctgcaca COM 10
cagtgacccacattcaggtgattacacacactcatacacagatccaTAC>---------<CCCCCCACCTCCACACACTCAAatgacctccccacgcccaaagctgcaca COM 9
cagtgacccacattcaggtgattacacacactcatacacagatccaTAC>-----<TGCCCCCCCCACCTCCACACACTCAAatgacctccccacgcccaaagctgcaca COM 5
cagtgacccacattcaggtgatt>--------------------------<GATCTTGCCCCCCCCACCTCCACACACTCAAatgacctccccacgcccaaagctgcaca COM 26
cagtgacccacattcaggtgattacacacactcatacacagatccaTAC>------------------<TCCACACACTCAAatgacctccccacgcccaaagctgcaca COM 18
cagtgacccacattcaggtgattacacacactcatacacagatccaTAC>-----------<CCCCACCTCCACACACTCAAatgacctccccacgcccaaagctgcaca COM 11
cagtgacccacattcaggtgattacacacactcatacacagatccaTAC>----------<CCCCCACCTCCACACACTCAAatgacctccccacgcccaaagctgcaca COM 10
cagtgacccacattcaggtgattacacacactcatacacagatccaTAC>---------<CCCCCCACCTCCACACACTCAAatgacctccccacgcccaaagctgcaca COM 9
cagtgacccacattcaggtgattacacacactcatacacagatccaTAC>---------<CCCCCCACCTCCACACACTCAAatgacctccccacgcccaaagctgcaca COM 9
cagtgacccacattcaggtgattacacacactcatacacagatccaTAC>--------<CCCCCCCACCTCCACACACTCAAatgacctccccacgcccaaagctgcaca COM 8
cagtgacccacattcaggtgattacacacactcatacacagatccaTACA>-------<CCCCCCCACCTCCACACACTCAAatgacctccccacgcccaaagctgcaca COM 7
cagtgacccacattcaggtgattacacacactcatacacagatccaTACA>------<CCCCCCCCACCTCCACACACTCAAatgacctccccacgcccaaagctgcaca COM 6
cagt>-------------------------------------------------------<CCCCCACCTCCACACACTCAAatgacctccccacgcccaaagctgcaca COM 55
cag>------------------------------------------------<TCTTGCCCCCCCCACCTCCACACACTCAAatgacctccccacgcccaaagctgcaca COM 48
cagtga>-------------------------------------------<GATCTTGCCCCCCCCACCTCCACACACTCAAatgacctccccacgcccaaagctgcaca COM 43
cagtgacccacattcaggtgattacacacactcatacacagatccaTAC>------------------------------------------<acgcccaaagctgcaca COM 43
cagtgacccacattcaggtgattacacacactcatacacagatccaTAC>-----------------------------------------<cacgcccaaagctgcaca COM 42
cagtgacccacattcaggtgattacacacactcatacacagatccaTAC>-----------------------------------------<cacgcccaaagctgcaca COM 42
```

**E**

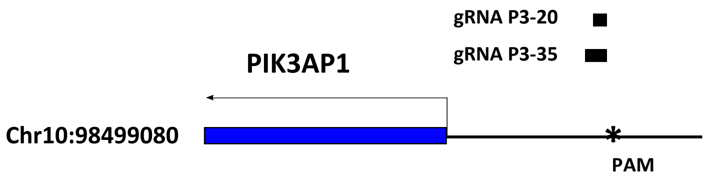


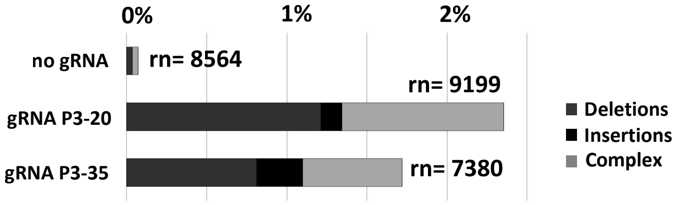


## no gRNA

PAM →

```
gtctcgaactactggcctcaagtgaTCCACCTGCCTCAGCCTCCCAAAGTGCTGGGATCaggtgtgagccaccaggcccagctgctttattatc WT  0
gtctcgaactactggcctcaagtgaTCCACCTGCCTCAGCCTCCCAAAGTGCTGGGATCaggtgtgagccacca-gcccagctgctttattatc DEL 1
gtctcgaactactggcctcaagtgaTCCACCTGCCTCAGCCT-CCAAAGTGCTGGGATCaggtgtgagccaccaggcccagctgctttattatc DEL 1
```

## gRNA P3-35

```
gtctcgaactactggcctcaagtgaTCCACCTGCCTCAGCCTCCCAAAGTGCTGGGATCaggtgtgagccaccaggcccagctgctttattatc WT  0
gtctcgaactactggcctcaagtgaTCCACCTGCCTCAGCCTCCCAAAGTGCT--GATCaggtgtgagccaccaggcccagctgctttattatc DEL 2
gtctcgaactactggcctcaagtgaTCCACCTGCCTCAGCCTCCCAAAGTGCT-GGATCaggtgtgagccaccaggcccagctgctttattatc DEL 1
gtctcgaactactggcctcaagtgaTCCACCTGCCTCAGCCTCCCA----------ATCaggtgtgagccaccaggcccagctgctttattatc DEL 10
gtctcgaactactggcctcaagtgaTCCACCTGCCTCAGCCTCCCAAAGTGCT---ATCaggtgtgagccaccaggcccagctgctttattatc DEL 3
gtctcgaactactggcctcaagtgaTCCACCTGCCTCA------------------ATCaggtgtgagccaccaggcccagctgctttattatc DEL 18
gtctcgaactactggcctcaagtgaTCCACCTGCCTCAGCCTCCCAAAGTGCTGGG--------------accaggcccagctgctttattatc DEL 14
gtctcgaactactggcctcaagtgaTCCACCTGCCTCAGCCTCCCAAA-------------gtgtgagccaccaggcccagctgctttattatc DEL 13
gtctcgaactactggcctcaagtgaTCCACCTGCCTCAGCCTCCCAA---------ATCaggtgtgagccaccaggcccagctgctttattatc DEL 9
gtctcgaactactggcctcaagtgaTCCACCTGCCTCAGCCTCCCAAA--------ATCaggtgtgagccaccaggcccagctgctttattatc DEL 8
gtctcgaactactggcctcaagtgaTCCACCTGCCTCAGCCTCCCAAAGTGCTG------ggtgtgagccaccaggcccagctgctttattatc DEL 6
gtctcgaactactggcctcaagtgaTCCACCTGCCTCAGCCTCCCAAAGT-----GATCaggtgtgagccaccaggcccagctgctttattatc DEL 5
gtctcgaactactggcctcaagtgaTCCACCTGCCTCAGCC---------------ATCaggtgtgagccaccaggcccagctgctttattatc DEL 15
gtctcgaactactggcctcaagtgaTCCACCTGCCTCAGCCTCCCAAAGTGCTGG--------gtgagccaccaggcccagctgctttattatc DEL 8
gtctcgaactactggcctcaagtgaTCCACCTGCCTCAGCCTCCCAAAGTGCTGGG---aggtgtgagccaccaggcccagctgctttattatc DEL 3
gtctcgaactactggcctcaagtgaTCCACCTGCCTCAGCCTCCCA---------------------------aggcccagctgctttattatc DEL 27
gtctcgaactactggcctcaagtgaTCCACCTGCCTCAGCCTCCCAAAGTGCTGGG------------------ggcccagctgctttattatc DEL 18
gtctcgaactactggcctcaagtgaTCCACCTGCCTCAGCCTCCCAAAGTGCTGGG-------------caccaggcccagctgctttattatc DEL 13
gtctcgaactactggcctcaagtgaTCCACCTGCCTCAGCCTC-------------ATCaggtgtgagccaccaggcccagctgctttattatc DEL 13
gtctcgaactactggcctcaagtgaTCCACCTGCCTCAGCCTCCCAAAGTGCTGG><TCaggtgtgagccaccaggcccagctgctttattatc INS 1
gtctcgaactactggcctcaagtgaTCCACCTGCCTCAGCCTCCCAAAGTGC><GGATCaggtgtgagccaccaggcccagctgctttattatc INS 1
gtctcgaactactggcctcaagtgaTCCACCTGCCTCAGCCTCCCAAAGTGCTGG><TCaggtgtgagccaccaggcccagctgctttattatc INS 1
gtctcgaactactggcctcaagtgaTCCACCTGCCTCAGCCTCCCAAAGTGCTGG><TCaggtgtgagccaccaggcccagctgctttattatc INS 3
gtctcgaactactggcctcaagtgaTCCACCTGCCTCAGCCTCCCAAAGTGCTGG>-<Caggtgtgagccaccaggcccagctgctttattatc COM 1
gtctcgaactactggcctcaagtgaTCCACCTGCCTCAGCCTCCCAAAGTGCTGGGAT>-----<gagccaccaggcccagctgctttattatc COM 5
gtctcgaactactggcctcaagtgaTCCACCTGCCTCAGCCTC>---------------------------<caggcccagctgctttattatc COM 27
gtctcgaactactggcctca>-----------------------------------<TCaggtgtgagccaccaggcccagctgctttattatc COM 35
gtctcgaactactggcctcaag>----------------------------------<Caggtgtgagccaccaggcccagctgctttattatc COM 34
```

## gRNA P3-20

```
gtctcgaactactggcctcaagtgaTCCACCTGCCTCAGCCTCCCAAAGTGCTGGGATCaggtgtgagccaccaggcccagctgctttattatc  WT 0
gtctcgaactactggcctcaagtgaTCCACCTGCCTCAGCCTCCCAAAGTGCT--GATCaggtgtgagccaccaggcccagctgctttattatc DEL 2
gtctcgaactactggcctcaagtgaTCCACCTGCCTCAGCCTCCCAA---------ATCaggtgtgagccaccaggcccagctgctttattatc DEL 9
gtctcgaactactggcctcaagtgaTCCACCTGCCTCAGCCTCCCAAAGTGCTGG------------gccaccaggcccagctgctttattatc DEL 12
gtctcgaactactggcctcaagtgaTCCACCTGCCTCAGCCTCCCAAAGTGCT---ATCaggtgtgagccaccaggcccagctgctttattatc DEL 3
gtctcgaactactggcctcaagtgaTCCACCTGCCTCAGCCTCCCAAAGTGC----ATCaggtgtgagccaccaggcccagctgctttattatc DEL 4
gtctcgaactactggcctcaagtgaTCCACCTGCCTCAGCCTCCCAAAGTGCT-GGATCaggtgtgagccaccaggcccagctgctttattatc DEL 1
gtctcgaactactggcctcaagtgaTCCACCTGCCTCAGCCTCCCAAAGTGC-----TCaggtgtgagccaccaggcccagctgctttattatc DEL 5
gtctcgaactactggcctcaagtgaTCCACCTGCCTCAGCCTCCCA----------ATCaggtgtgagccaccaggcccagctgctttattatc DEL 10
gtctcgaactactggcctcaagtgaTCCACCTGCCTCAGCCTCCCAAAGTGCTGG--------gtgagccaccaggcccagctgctttattatc DEL 8
gtctcgaactactggcctcaagtgaTCCACCTGCCTCAGCCTCCCAAA--------ATCaggtgtgagccaccaggcccagctgctttattatc DEL 8
gtctcgaactactggcctcaagtgaTCCACCTGCCTCAGCCTCCCAAAGTGCTGGG-------gtgagccaccaggcccagctgctttattatc DEL 7
gtctcgaactactggcctcaagtgaTCCACCTGCCTCAGCCTCCCAAA-------GATCaggtgtgagccaccaggcccagctgctttattatc DEL 7
gtctcgaactactggcctcaagtgaTCCACCTGCCTCAGCCTCCCAAAGTGCTG------ggtgtgagccaccaggcccagctgctttattatc DEL 6
gtctcgaactactggcctcaagtgaTCCACCTGCCTCAGCCTCCCAAAGTG------------------caccaggcccagctgctttattatc DEL 18
gtctcgaactactggcctcaagtgaTCCACCTGCCTCAGCCTCCCAAAGTGCTGGG--------------accaggcccagctgctttattatc DEL 14
gtctcgaactactggcctcaagtgaTCCACCTGCCTCAGCCTCC--------------Caggtgtgagccaccaggcccagctgctttattatc DEL 14
gtctcgaactactggcctcaagtgaTCCACCTGCCTCAGCCTCCCAAAGTGCTGG-----ggtgtgagccaccaggcccagctgctttattatc DEL 5
gtctcgaactactggcctcaagtgaTCCACCTGCCTCAGCCTCCCAAAGTGCTGGG--Caggtgtgagccaccaggcccagctgctttattatc DEL 2
gtctcgaactactggcctcaagtgaTCCACCTGCCTCAGCCTCCCAAAGTGCTGG><TCaggtgtgagccaccaggcccagctgctttattatc INS 1
gtctcgaactactggcctcaagtgaTCCACCTGCCTCAGCCTCCCAAAGTGCTGG><TCaggtgtgagccaccaggcccagctgctttattatc INS 1
gtctcgaactactggcctcaagtgaTCCACCTGCCTCAGCCTCCCAAAGTGCTG><ATCaggtgtgagccaccaggcccagctgctttattatc INS 3
gtctcgaactactggcctcaagtgaTCCACCTGCCTCAGCCTCCCAAAGTGCTGG><TCaggtgtgagccaccaggcccagctgctttattatc INS 2
gtctcgaactactggcctcaagtgaTCCACCTGCCTCAGCCTCCCAAAGTGCTGG><TCaggtgtgagccaccaggcccagctgctttattatc INS 1
gtctcgaactactggcctcaagtgaTCCACCTGCCTCAGCCTCCCAAAGTGCT>------------------<aggcccagctgctttattatc COM 18
gtctcgaactactggcctcaagtgaTCCACCTGCCTCAGCCTCC>------------<Caggtgtgagccaccaggcccagctgctttattatc COM 12
gtctcgaactactggcctcaagtgaTCCACCTGCCTCAGCCTCC>------------<Caggtgtgagccaccaggcccagctgctttattatc COM 12
```
