# Supplementary material for: CORALINA: a universal method for the generation of gRNA libraries for CRISPR-based screening
Source: BMC Genomics. 2016 Nov 14;17:917. doi: 10.1186/s12864-016-3268-z (PMC5109649; doi:10.1186/s12864-016-3268-z)
Supplement: Additional file 5: Table S1. — Quantification of NGS read number and GC content for the four analysed NGS sequencing samples. (PDF 19 kb) [file 12864_2016_3268_MOESM5_ESM.pdf]

CORALINA Library	Trimmed Reads	Trimmed Reads (18+)		Trimmed Reads (unique)		GC Content
Human L1	867098	858459	99.0%	737392	85.9%	58.6
Human L2	361683	336219	93.0%	294305	87.5%	61.5
Human L3	311074	283926	91.3%	262438	92.4%	62.7
Mouse	1394121	1370644	98.3%	1087170	79.3%	47.4
